# Promoting men’s awareness, self-examination, and help-seeking for testicular disorders: a systematic review of interventions

**DOI:** 10.12688/hrbopenres.12837.2

**Published:** 2018-07-06

**Authors:** Mohamad M. Saab, Martin Davoren, Aileen Murphy, David Murphy, Eoghan Cooke, Margaret Landers, Serena Fitzgerald, Noel Richardson, Michael Rovito, Christian Von Wagner, Mike Murphy, Darren Dahly, Josephine Hegarty

**Affiliations:** 1 School of Nursing and Midwifery, University College Cork, Cork, Ireland; 2 School of Public Health, University College Cork, Cork, Ireland; 3 Sexual Health Centre, Cork, Ireland; 4 Department of Economics, University College Cork, Cork, Ireland; 5 Department of Computer Science, University College Cork, Cork, Ireland; 6 Department of Science and Health, Institute of Technology Carlow, Carlow, Ireland; 7 College of Health and Public Affairs, University of Central Florida, Orlando, Florida, USA; 8 Research Department of Behavioural Science and Health, University College London, London, UK; 9 School of Applied Psychology, University College Cork, Cork, Ireland; 10 Health Research Board Clinical Research Facility, University College Cork, Cork, Ireland

**Keywords:** Awareness, health promotion, help-seeking, men’s health, systematic review, testicular cancer, testicular diseases, testicular self-examination

## Abstract

**Background:** Testicular cancer (TC) is commonly diagnosed among men aged 15-40 years. The incidence of TC is on the rise. Benign testicular disorders such as testicular torsion and epididymitis can lead to testicular ischemia, sepsis, and infertility if left untreated. This systematic review aims to evaluate the effectiveness of studies promoting men’s knowledge and awareness of testicular disorders and/or self-examination, behaviours and/or intentions to examine their testes, and help-seeking behaviours and/or intentions for testicular symptoms.

**Methods:** Academic Search Complete, Medline, CINAHL, PsychINFO, ERIC, the Cochrane Library, the World Health Organisation International Clinical Trials Registry Platform, Clinicaltrials.gov, Grey Literature Report, and Open Grey were searched for studies published between November 2014 and April 2018. The methodological quality and level of evidence per outcome were assessed.

**Results: **There were five papers included: two were experimental studies, two were systematic reviews, and one was an integrative review. The majority of the reviewed interventions were successful in increasing men’s awareness of TC and self-examination. Examples include a television show featuring a celebrity with TC, a university campaign, and interactive educational sessions. The impact of the reviewed interventions on health beliefs (i.e. perceived susceptibility, severity, benefits, barriers, and self-efficacy) varied across the reviewed literature. Studies promoting help-seeking for testicular symptoms and awareness of benign testicular disorders were lacking.

**Conclusions: **This review highlights the importance of evaluating educational interventions aimed at younger men, whilst raising their awareness of testicular disorders and increasing their help-seeking intentions for testicular symptoms. Given the lack of consensus around scheduled testicular self-examination among younger men, clinicians are encouraged to instruct men to familiarise themselves with the look and feel of their own testes and to seek timely medical attention for abnormalities.

**Registration:** The review protocol was registered with the International Prospective Register of Systematic Reviews (PROSPERO) under the registration number CRD42018093671.

## Introduction

According to the National Cancer Institute, testicular cancer (TC) is most commonly diagnosed among men aged 15 to 40 years. The incidence of TC has doubled globally over the past 40 years and is highest in Western and Northern European countries, Australia, and North America ^1, 2^. According to the National Cancer Registry Ireland, 90% of TC cases and 85% of TC deaths in Ireland occur among men younger than 50 years. Furthermore, the incidence of TC in Ireland is increasing by 2.3% annually. A unilateral painless testicular mass is a classical sign of TC. Testicular pain, back pain, cough, haemoptysis, and headaches can be warning signs of metastatic TC ^3, 4^.

Benign testicular disorders (BTDs) can also have a negative impact on a man’s health. Epididymo-orchitis, often contracted sexually by men younger than 50 years, is known to be the primary cause of acute scrotal pain. This infection can cause sepsis and infertility if not diagnosed and managed promptly ^5^. Testicular torsion is characterised by severe scrotal pain, oedema, nausea, and vomiting, and can lead to testicular ischemia and necrosis if testicular perfusion is not restored within 6 hours of the onset of pain ^5– 7^. The severity of these conditions highlight the potential role of testicular awareness and testicular self-examination (TSE) in detecting TC as well as BTDs ^8, 9^.

A systematic review of 25 studies exploring men’s awareness of TC and TSE found that men were unaware of TC risk factors, signs and symptoms, and treatments, and that very few reported performing TSE ^10^. These findings were echoed by Roy and Casson, who explored the awareness, knowledge, and attitudes regarding TC and TSE of 150 men in Northern Ireland ^11^. This study found that only 39% of participants correctly identified the TC at-risk age group, and only 17% were aware of TSE ^11^.

Very little recent evidence exists in relation to BTD awareness. Saleem *et al.* explored men’s awareness of BTDs in Pakistan and found that 78.8% of participants were unaware of the symptoms of BTDs, 73.6% reported that BTDs were considered taboo, and 29.8% did not intend to perform TSE ^12^. Yap *et al.* surveyed Irish parents (n=242) about their awareness and help-seeking for testicular torsion ^13^. This study found that parents who were aware of torsion were four times more likely to seek immediate help (OR, 4.2; 95% CI, 1.4-12.2; p<0.01) than those who lacked awareness. Moreover, participants who correctly identified the timeframe for help-seeking were three times more likely to seek immediate help than those who did not know the timeframe (OR, 3.0; 95% CI, 0.85-10.8; p=0.08) ^13^.

There is no consensus regarding the effectiveness of monthly TSE in detecting testicular disorders early ^14^, which resulted in different recommendations regarding this practice globally. For instance, the U.S. Preventive Services Task Force opposes this practice ^15^, whereas Cancer Research UK and the Irish Cancer Society encourage men to check their testes and report any abnormalities to a healthcare professional. TSE proponents were critical of the decision made by U.S. Preventive Services Task Force and stated that TSE has potential benefits beyond the early detection of TC such as familiarising men with their own testes and helping detect TC and BTDs early ^16^. McGuinness *et al.* highlighted that public health initiatives promoting TSE were linked to early TC diagnosis and smaller tumour size at diagnosis ^17^. Furthermore, in their cost-utility analysis of TC and TSE, Aberger *et al.* found that a 2.4 to 1 cost-benefit ratio was established for early-onset versus advanced TC ^18^, which emphasises the importance of raising men’s awareness of diseases of the testes.

Saab *et al.* systematically reviewed evidence from 11 experimental studies (2004–2014) promoting men’s awareness of TC and TSE, and increasing their TSE intentions and behaviours ^19^. Saab *et al.* also conducted an integrative review of the literature on BTD awareness (1985–2015) ^20^. Despite men’s lack of awareness of BTDs and their intentions to delay help-seeking for symptoms of testicular disease, none of these reviews included studies that aimed at promoting men’s awareness of BTDs and/or increasing their intentions to seek help for testicular symptoms. The present review builds upon the search, screening, and output from both reviews ^19, 20^. Of note, there is no gold standard for the frequency of updating structured reviews ^21^. However, biennial review updates are recommended by the Cochrane Library.

### Objectives

The aim of this systematic review is to evaluate the effectiveness of experimental studies, and the findings of structured reviews of experimental studies promoting men’s knowledge and awareness of testicular disorders and/or self-examination, behaviours and/or intentions to examine their testes, and help-seeking behaviours and/or intentions for testicular symptoms. The primary outcomes of this review are presented below using the PICOS (participants, interventions, comparisons, outcomes, and study design) framework ( http://handbook-5-1.cochrane.org/):

Primary outcomes:

1. The effect of intervention on men’s knowledge and awareness of testicular disorders and/or self-examination, compared to baseline and/or control conditions (i.e. alternative intervention or no intervention).

2. The effect of intervention on men’s behaviours and/or intentions to examine their testes, compared to baseline and/or control conditions (i.e. alternative intervention or no intervention).

3. The effect of intervention on men’s help-seeking behaviours and/or intentions for testicular symptoms.

Due to the anticipated dearth of literature on testicular disorders, structured reviews of experimental studies and secondary outcomes such as measures of benefits and/or harms, economic evaluations, and process evaluations were also considered.

## Methods

### Protocol and registration

This systematic review was guided by the Cochrane Handbook for Systematic Reviews of Interventions ( http://handbook-5-1.cochrane.org/), and reported using the Preferred Reporting Items for Systematic Reviews and Meta-Analysis (PRISMA) checklist ^22^ ( Supplementary File 1). The review questions and methods were predetermined and were not amended during the review process. The review protocol was registered with the International Prospective Register of Systematic Reviews (PROSPERO) under the registration number CRD42018093671.

### Eligibility criteria

Studies were eligible for inclusion if they used an experimental or structured review design and were conducted among men who did not have a diagnosis of a testicular disorder. Studies addressing primary and/or secondary outcomes and studies evaluating the effect of intervention(s) compared to baseline and/or control conditions were included. The full inclusion criteria are reported in Table 1 using the PICOS framework.

**Table 1.** Review inclusion criteria using the PICOS framework.

**Participants**	Men without a diagnosis of a testicular disorder
**Interventions**	Educational/health promotion intervention/programme
**Comparisons**	The effect of intervention compared to baseline and/or control conditions i.e. alternative intervention(s) or no intervention
**Outcomes** **(primary)**	(i) Knowledge and awareness of testicular disorders and/or self-examination (ii) Behaviours and/or intentions to examine/feel own testes (iii) Help-seeking behaviours and/or intentions for testicular symptoms
**Study design**	Any experimental design (i.e. randomised controlled trial, non-randomised controlled trial, pre-post study design with one or more groups, and post-test only study design with one or more groups) and structured reviews of interventions (i.e. systematic and integrative reviews)

Men with a diagnosis of a testicular disorder, studies with women only, and studies where findings from men and women are indistinguishable were excluded. Additionally, quantitative descriptive studies, qualitative studies, opinion papers, and conference abstracts were not eligible for inclusion. Theses and dissertations were also excluded because the merit of their use in systematic reviews is questionable ^23^.

### Information sources and search strategy

The following electronic databases were searched on April 13 ^th^ 2018: Academic Search Complete, Medline, CINAHL, PsycINFO, ERIC, and The Cochrane Library. In addition, eligible studies were sought from trial registries including the World Health Organisation International Clinical Trials Registry Platform (ICTRP) and Clinicaltrials.gov. The grey literature (i.e. the Grey Literature Report and Open Grey) and reference lists of eligible papers were also reviewed for eligible papers. The search was limited to records published in English between November 1 ^st^ 2014 (the date of the last search in the review by Saab *et al.* ^16^) and April 30 ^th^ 2018.

The following keywords were searched on title and abstract using Boolean operators “OR” and “AND”: “testicular disease*” OR “testicular disorder*” OR “testicular cancer” OR “testicular neoplas*” OR “testicular tumor*” OR “testicular tumour*” OR “testicular malignan*” OR “benign testicular disorder*” OR “benign testicular disease*” OR “testicular torsion” OR epididymitis OR orchitis OR epididymo-orchitis OR hydrocele OR varicocele OR spermatocele OR “testicular symptom*” OR “testicular pain” OR “testicular lump*” OR “testicular swelling” OR “scrot* symptom*” OR “scrot* pain” OR “scrot* lump*” OR “scrot* swelling” AND knowledge OR awareness OR practice* OR self-exam* OR “self exam*” OR feel* OR screen* OR “early detect*” OR help-seeking OR “help seeking” OR “help-seeking intention*” OR “help seeking intention*” OR “help-seeking behavior*” OR “help-seeking behaviour*” OR “help seeking behavior” OR “help seeking behaviour” AND intervention* OR inform* OR educat* OR “health education” OR “health promotion” OR trial* OR experiment* OR stud* OR program*.

### Study selection and data extraction

Records identified from electronic databases, trial registries, and grey literature searches were exported to a software package for reference management (EndnoteX8). Duplicates were then deleted and the records were transferred to Covidence, an online service use by Cochrane reviewers to facilitate screening and data extraction.

All records were screened on title and abstract. Following the exclusion of irrelevant records, the full-text of potentially eligible studies was obtained for further screening. Title, abstract, and full-text screenings were conducted by two independent reviewers (M.M.S. and J.H.). Screening conflicts were resolved either by consensus or a third reviewer.

A standardised extraction table was used to extract data from experimental studies ^19, 20^. Data were extracted by one reviewer (M.M.S.) and cross-checked for accuracy by a second reviewer (J.H.). The following data were extracted: author(s) and year; aim(s); country, setting and funding; participants; design and theoretical underpinning; intervention(s); outcome(s) and data collection; and findings presented according to the review questions. As for structured reviews, a separate data extraction table was designed by two experienced reviewers (M.M.S. and J.H.) to include the following: author(s), year, and country; aim(s); review type and funding; eligibility criteria; data sources; study selection and data extraction; quality appraisal; and study characteristics and findings.

### Quality and level of evidence assessment

The Quality Assessment Tool (QAT), developed by the Effective Public Health Practice Project (EPHPP), was used to appraise the methodological quality of experimental studies ( http://www.nccmt.ca/knowledge-repositories/search/14). This tool is recommended in the Cochrane Handbook for Systematic Reviews of Interventions ( http://handbook-5-1.cochrane.org/). The quality of the studies was judged as either Strong, Moderate, or Weak based on the following criteria: selection bias; study design; confounders; blinding; data collection methods; withdrawal and dropouts; intervention integrity; and analyses.

The Grading of Recommendations Assessment, Development and Evaluation (GRADE) tool was then used to assess the level of evidence per outcome ^25^. “The quality of the evidence was assessed in terms of methodological limitations, heterogeneity and/or inconsistency of findings, indirectness of evidence, imprecision of results, and publication bias” (p. 475) ^19^. Eligible studies were included regardless of their methodological quality in order to minimise the risk of reporting bias.

The AMSTAR 2 measurement tool was used to assess the methodological quality of structured reviews ^25^. The domains within this tool address 16 key questions in relation to: using PICO to guide the review question and eligibility criteria; reporting on the review methods; explaining the choice of study designs; conducting the literature search; selecting and extracting data in duplicate; justifying and describing study inclusion and exclusion; assessing the risk of bias; reporting on sources of funding; conducting a meta-analysis; discussing study heterogeneity; and reporting conflict(s) of interest ^21^.

### Data synthesis

A meta-analysis with summary measures of treatment effect using weighted/standard mean difference, risk/odds ratios, and 95% confidence was planned using RevMan 5, if the included studies were sufficiently homogenous. However, the included studies were heterogeneous in terms of intervention format, data collection, and participant allocation; therefore, findings from the reviewed studies were synthesised meta-narratively.

## Results

### Study selection

**Figure 1.**
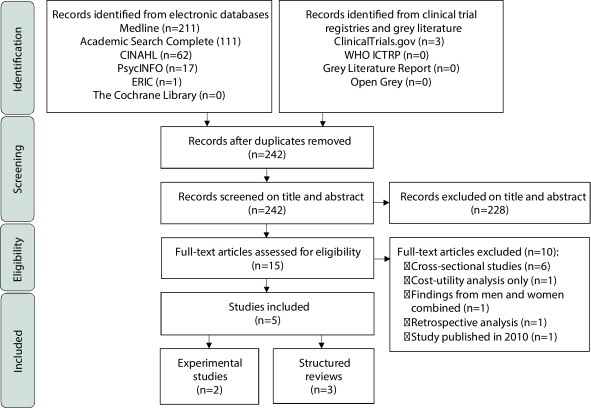
Flow diagram detailing study identification, screening, and selection process.

A total of 405 records were identified from electronic databases, clinical trial registries, and grey literature searches. No additional records were identified from reference list checks. Following the exclusion of duplicates, 242 records were screened on title and abstract. Of those, 15 full-text articles were assessed for eligibility and 10 were excluded, with the majority being cross-sectional studies (n=6). As a result, five papers were included in the present review; two were experimental studies and three were structured reviews. The full study selection process and reasons for exclusion are presented in Figure 1.

### Study characteristics

The two experimental studies were conducted in Turkey and were underpinned by the Health Belief Model ^26, 27^. Both studies explored the awareness of TC and TSE, TSE behaviours, and perceived susceptibility, severity, benefits of TSE, barriers to TSE, and self-efficacy ^26, 27^. Sample sizes were n=96 ^26^ and n=174 ^27^. Data were collected from patient care personnel (i.e. care assistants) ^26^ and university students ^27^. Akar and Bebiş used a prospective, randomized, controlled intervention design ^26^, whereas Pour *et al.* conducted a quasi-experimental follow-up study ^27^.

Of the three structured reviews, two were systematic reviews ^19, 28^, and one was an integrative review ^20^. The review by Rovito *et al.* included 10 studies ^28^, and the reviews by Saab *et al.* included 11 and 4 studies, respectively ^19, 20^. Rovito *et al.* addressed TSE behaviours only ^28^, Saab *et al.* explored TC and TSE awareness and TSE intentions and behaviours ^19^, and Saab *et al.* explored awareness of BTDs ^20^.

### Quality and level of evidence assessment

Both experimental studies had a “Weak” overall quality rating since both failed to address confounders and blinding ^26, 27^. Items in relation to selection bias, study design, and withdrawal and dropout were rated as “Poor” in the study by Pour *et al.* ^27^ ( Table 2).

**Table 2.** Quality appraisal of experimental studies using the Quality Assessment Tool (QAT).

QAT items	Akar and Bebiş (2014)	Pour *et al*. (2018)
1. Selection bias	Good	Poor
2. Study design	Good	Poor
3. Confounders	Poor	Poor
4. Blinding	Poor	Poor
5. Data collection methods	Good	Good
6. Withdrawals and dropouts	Good	Poor
7. Intervention integrity		
*(Q1) Percentage of intervention recipients*	80–100%	80–100%
*(Q2) Consistency measured*	Can’t tell	Can’t tell
*(Q3) Risk for contamination*	Can’t tell	Can’t tell
8. Analysis		
*(Q1) Unit of allocation*	Individual	Individual
*(Q2) Unit of analysis*	Individual	Individual
*(Q3) Appropriate statistical methods*	Yes	Yes
*(Q4) Intention to treat*	Yes	Yes
**OVERALL RATING**	**WEAK**	**WEAK**

The quality of evidence was “Very Low” for two outcomes, namely TC and TSE awareness and TSE behaviours, and “Low” for health belief in relation to TC and TSE. These ratings were attributed to a number of limitations including the lack of blinding and allocation concealment, lack of sample size calculation and power analysis, and lack of effect size and magnitude of effect measures ( Table 3).

**Table 3.** Level of evidence assessment per review outcome.

Outcomes	Number of participants (studies)	Risk of bias	Inconsistency	Indirectness	Imprecision	Publication bias	Overall quality (GRADE)
TC and TSE awareness	270 (2 studies)	Yes	No	Yes	Yes	No	+OOO Very low
TSE behaviours	270 (2 studies)	Yes	No	Yes	Yes	No	+OOO Very low
Health beliefs	270 (2 studies)	Yes	No	No	Yes	No	++OO Low

TC, testicular cancer; TSE, testicular self-examination.

As for the structured reviews, none mentioned using PICO to guide the research questions or inclusion criteria and none reported whether methods were established prior to conducting the reviews. In addition, none of the three reviews reported on the sources of funding for the included studies ^19, 20, 28^. Rovito *et al.* did not list the search terms, justify study exclusion, or report on heterogeneity in the results ^28^ ( Table 4).

**Table 4.** Quality appraisal of integrative and systematic reviews using the AMSTAR 2 instrument.

AMSTAR 2 questions	Rovito *et al*. (2015)	Saab *et al*. (2016a)	Saab *et al*. (2016b)
1. Did the research questions and inclusion criteria for the review include the components of PICO?	No	No	No
2. Did the report of the review contain an explicit statement that the review methods were established prior to the conduct of the review and did the report justify any significant deviations from the protocol?	No	No	No
3. Did the review authors explain their selection of the study designs for inclusion in the review?	Yes	Yes	Yes
4. Did the review authors use a comprehensive literature search strategy?	No	Partial Yes	Partial Yes
5. Did the review authors perform study selection in duplicate?	Yes	Yes	Yes
6. Did the review authors perform data extraction in duplicate?	No	Yes	Yes
7. Did the review authors provide a list of excluded studies and justify the exclusions?	No	Yes	Yes
8. Did the review authors describe the included studies in adequate detail?	Yes	Yes	Yes
9. Did the review authors use a satisfactory technique for assessing the risk of bias (RoB) in individual studies that were included in the review?	Yes	Yes	Yes
10. Did the review authors report on the sources of funding for the studies included in the review?	No	No	No
11. If meta-analysis was performed did the review authors use appropriate methods for statistical combination of results?	NA	NA	NA
12. If meta-analysis was performed, did the review authors assess the potential impact of RoB in individual studies on the results of the meta-analysis or other evidence synthesis?	NA	NA	NA
13. Did the review authors account for RoB in individual studies when interpreting/discussing the results of the review?	Yes	Yes	Yes
14. Did the review authors provide a satisfactory explanation for, and discussion of, any heterogeneity observed in the results of the review?	No	Yes	Yes
15. If they performed quantitative synthesis did the review authors carry out an adequate investigation of publication bias (small study bias) and discuss its likely impact on the results of the review?	NA	NA	NA
16. Did the review authors report any potential sources of conflict of interest, including any funding they received for conducting the review?	Yes	Yes	Yes

NA, not applicable.

### Synthesis of results

Results of experimental studies and structured reviews are presented in Table 5 and Table 6, respectively.

**Table 5.** Data extraction table for experimental studies.

Author(s) & year	Aim(s)	Country, setting & funding	Participants	Design & theoretical underpinning	Intervention(s)	Outcome(s) and data collection	Findings *
Akar and Bebiş (2014)	“To assess health beliefs and knowledge of testicular cancer (TC) and testicular self-examination (TSE) and the effectiveness of TC and TSE training for patient care staff” (p.966)	Turkey Hospital Funding not reported (NR)	n=96 male patient care personnel (assistants of healthcare professionals) randomly assigned to two groups, *Group 1* (n=48; interactive education group) and *Group 2* (n=48; pamphlet group)	Prospective, randomized, controlled intervention study Health Belief Model (HBM)	*Group 1*: 45-minute PowerPoint presentation on TC and TSE (cases of two patients, 5-min video depicting how patients did not know how to perform TSE, messages on the importance of not being afraid of TC and TSE) *Group 2*: Pamphlet on TC and TSE	Data collected at pre-test and post-test (12 weeks) using a 51-item researcher-designed questionnaire: 25 items assessed demographics, TC knowledge and practice. 26 items comprised five Champion Health Belief Model (CHBM) sub- dimensions, perceived: Susceptibility (5) Severity (7) Benefits of TSE (3) Barriers to TSE (5) Self-efficacy (6)	**(Q1)** 54.1% (n=52) were unaware of TC and TSE at pre-test. Knowledge increased for both groups at post-test (p=0.001), with *Group 1* having greater knowledge (p=0.005) **(Q2)** 5.2% (n=5) reported practicing TSE at pre-test. At post-test, 83.3% (n=40) in *Group 1* and 54.2% (n=26) in *Group 2* reported practicing TSE (p=0.002) **(Q3)** Not reported (NR) **(Q4)** Perceived susceptibility, severity, benefits, and confidence increased (p=0.001) and perceived barriers decreased (p=0.001) at post-test for both groups
Pour *et al*. (2018)	“To evaluate the efficacy of TSE education on knowledge, performance, and health beliefs of Turkish young men” (p.398)	Turkey University No funding	n=174 male nursing and nutrition-dietetic students randomly assigned into 12 groups (12–18 students/group)	Quasi- experimental follow-up study design HBM	Each group was given education about TC and TSE using PowerPoint presentation, video, pamphlet, and question-answer interaction	Data collected using a research designed questionnaire with socio- demographic questions and questions assessing knowledge, attitudes, and behaviours toward TC and TSE (pre-test only), The Turkish version of CHBM scale with five sub- dimensions was administered at pre- and 3 months post- test: Sensitiveness (5) Caring/ seriousness (7) Benefits (3) Obstacles (5) Self-effectiveness/ confidence (6)	**(Q1)** At pre-test, 82.8% (n=144) heard about TC, 40.8% (n=71) were not informed about TC, 54.5% (n=95) did not hear about TSE, and 72.4% (n=126) were not educated about TSE **(Q2)** At pre-test, 76.5% (n=133) did not perform TSE, 81% (n=141) thought that TSE should be done, and 50.5% (n=88) did not know how to perform TSE **(Q3)** NR **(Q4)** Perceived sensitiveness decreased (11.27/25±3.6 pre-test vs. 10.42±4.55 post-test; p=0.01), benefits increased (10.68/15±2.8 pre-test vs. 11.74±2.41 post-test; p=0.003), and seriousness, obstacles, and self- effectiveness did not vary significantly at post-test

*Findings presented according to the review questions as follows: **(Q1)** Knowledge and awareness of testicular disorders and/or self-examination; **(Q2)** Behaviours and/or intentions to examine/feel their testes; **(Q3)** Help-seeking behaviours and/or intentions for testicular symptoms; **(Q4)** Secondary outcomes in relation to measures of benefits/harms, economic evaluations, process evaluations, and other testicular-related measures. CHBM, Champion’s health belief model; HBM, health belief model; NR, not reported; TC, testicular cancer; TSE, testicular self-examination.

**Table 6.** Data extraction table for integrative and systematic reviews.

Author(s),year & country	Aim(s)	Review type & funding	Eligibility criteria	Data sources	Study selection & data extraction	Quality appraisal	Study characteristics & Findings *
Rovito *et al*. (2015) USA	To organise and assess evidence from interventions promoting testicular self-examination (TSE) performance among at-risk men	Systematic review No funding	*Inclusion:* Peer reviewed, English language, experimental studies *Exclusion:* Studies with participants who have sought care for a testicular problem(s), studies on the aetiology and treatments of testicular cancer (TC), interventions solely aimed to increase TSE knowledge, awareness, and intentions	Ovid Medline, CINAHL, PsycInfo, All EBM Reviews, Ovid Healthstar, ERIC, and Google Scholar were searched	Title, abstract, and full-text screenings conducted by three reviewers. Data extracted: authors, quality, sample size, intervention design, theoretical framework, primary outcomes, significance level, and weaknesses	Downs and Black’s (1998) checklist used. Nine studies were of “Average” quality and one was of “High” quality	n=10 experimental studies included. Sample sizes ranged between 48 and 835. 6 studies were underpinned by theory **(Q1)** Not reported (NR) **(Q2)** 3 studies did not achieve statistical significance: film vs. print media; promotional vs. no promotional materials; and print material and shower cards vs. video on TSE and shower cards vs. no information **(Q3)** NR **(Q4)** NR
Saab *et al*. (2016a) Ireland	To extract and analyse evidence from studies that explored males’ awareness of benign testicular disorders (BTDs)	Integrative review No funding	*Inclusion:* Descriptive and experimental studies and structured reviews published in English in peer-reviewed journals (1985–2015). *Exclusion:* Papers with an overview of BTDs, TC, men with BTDs, women only, opinion papers and epidemiological studies	CINAHL, Medline, PsychINFO, and PubMed were searched and reference lists of eligible studies were checked	Title, abstract, and full-text screenings conducted independently by two reviewers. Data extracted: Citation, aim, country and setting, population, design, instruments, and findings	A tool developed to appraise the quality of cross-sectional studies in previous reviews was used. The quality of all four studies was ranked as “Moderate”	n=4 cross-sectional studies included. No experimental studies on BTDs sourced
Saab *et al*. (2016b) Ireland	To review studies conducted to enhance men’s knowledge and awareness of testicular cancer (TC) and testicular self-examination (TSE) and increase their TSE behaviours and intentions	Systematic review No funding	*Inclusion:* Experimental studies, published in English (2004–2014), and included men only *Exclusion:* Descriptive studies, opinion papers, studies with women only, reviews, and conference abstracts	Medline, CINAHL, and EMBASE were searched and reference lists of eligible studies were checked	Title, abstract, and full-text screenings conducted independently by two reviewers. Data extracted: reference and year, country and setting, design and theoretical underpinning, data collection, findings, and quality appraisal	Quality Assessment Tool (QAT) used. 6 studies were rated as “Weak”, 4 as “Moderate”, and 1 as “Strong.” The level of evidence per outcome assessed using the GRADE tool and was “Very Low” for TC awareness and “Low” for TSE awareness, intentions, and practices	n=11 experimental studies included. Sample sizes ranged between 74 and 874. 6 studies were underpinned by theory **(Q1)**10 studies addressed TC knowledge. All but 1 study (print material and shower cards vs. video on TSE and shower cards vs. no information) increased TC knowledge significantly. 7 studies addressed TSE knowledge, which ranged between 4% (n=3) and 53.2% (n=83) at baseline **(Q2)** 6 studies addressed TSE intentions. All but one study (Implementation Intentions statements) significantly increased TSE intentions. 7 studies addressed TSE behaviours, which increased significantly in all 7 studies **(Q3)** NR **(Q4)** NR

*Findings presented according to the review questions as follows: **(Q1)** Knowledge and awareness of testicular disorders and/or self-examination; **(Q2)** Behaviours and/or intentions to examine/feel their testes; **(Q3)** Help-seeking behaviours and/or intentions for testicular symptoms; **(Q4)** Secondary outcomes in relation to measures of benefits/harms, economic evaluations, and process evaluations. BTDs, benign testicular disorders; NR, not reported; TC, testicular cancer; TSE, testicular self-examination.

### Awareness of testicular disorders and self-examination

Three of the reviewed papers addressed men’s awareness of TC and TSE ^19, 26, 27^. However, interventions promoting awareness of BTDs were lacking.

Akar and Bebiş conducted a prospective randomised controlled trial comparing the effect of two interventions (45-minute interactive PowerPoint presentation (Group 1) and pamphlets (Group 2)) on men’s (n=96) awareness of TC and TSE and assessing their health beliefs in relation to TSE ^26^. Approximately half of the participants (54.1%, n=52) were unaware of TC and TSE at pre-test. However, knowledge increased significantly at 3 months post-test for both groups (p=0.001), but was significantly higher among Group 1 than in Group 2 (p=0.005). Similarly, Pour *et al.* conducted a quasi-experimental follow-up study to evaluate the effectiveness of TC and TSE education (i.e. PowerPoint, video, pamphlet, and question and answer sessions) on men’s (n=174) knowledge of TC and TSE, TSE behaviours, and health belief in relation to TSE ^27^. Of note, data in relation to TC and TSE awareness were collected at pre-test only. The majority of the participants (82.8%, n=144) reported that they have heard of TC; however, only 40.8% (n=71) were informed about this malignancy. Likewise, almost half of the participants were unaware of TSE (54.5%, n=95) and 72.4% (n=126) were not educated about this practice ^27^.

Saab *et al.* reviewed evidence from 11 experimental studies promoting men’s knowledge and awareness of TC and TSE and increasing their TSE behaviours and intentions ^19^. Some of the interventions addressed knowledge of TC and TSE at pre-test only. Baseline knowledge of TC risk factors ranged between 7.75% ^29^ and 50.6% ^30^. Similarly, knowledge of TSE ranged between 4% ^31^ and 53.2% ^30^.

The majority of the studies reviewed by Saab *et al.* were successful in increasing knowledge and awareness of TC and/or TSE ^19^. For instance, TC knowledge increased significantly as a result of a video on TC filmed in the American Sign Language (p<0.05) ^32^; shower gel sachets, waterproof stickers, and posters (p=0.014) ^33^; a TC educational video (p<0.001) ^34^; and a TC university campaign (p<0.001) ^35^. Furthermore, awareness of TSE increased significantly following a multimodal intervention comprising lectures, discussions, role-plays, posters, pamphlets, booklets, and screening sessions (p<0.001) ^31^. Interventions that significantly increased men’s awareness of both TC and TSE included: a television show featuring a celebrity with TC (p<0.001) ^36^; TC and TSE factsheets and testimonies from fictitious patients (p<0.001) ^37^; and TC facts and TSE advice (p=0.004) ^38^. On the other hand, a three-armed intervention comparing the effect of print material and shower cards versus video on TSE and shower cards versus no information, did not identify a significant difference in increase in knowledge of TC and TSE (p=0.7) ^39^.

### Behaviours and intentions to perform testicular self-examination

TSE behaviours and/or intentions were explored in four of the reviewed papers ^19, 26– 28^. Pour *et al.* measured TSE behaviours at pre-test only and found that 50.5% (n=88) of participants did not know how to perform TSE and 76.5% (n=126) did not perform TSE ^27^. However, 81% (n=141) believed that TSE should be done ^27^. Only 5.2% (n=5) of participants in the study by Akar and Bebiş reported performing TSE at pre-test ^26^. This increased significantly to 83.3% (n=40) among Group 1 (45-minute interactive PowerPoint presentation) and 54.2% (n=26) among Group 2 (pamphlets) three months post-test (p=0.002) ^26^.

Of the 11 studies reviewed by Saab *et al.*, six explored TSE intentions and/or behaviours ^19^. The following four interventions significantly increased men’s intentions to perform TSE: a television show featuring a celebrity with TC (p<0.001) ^36^; TC and TSE factsheets and testimonies from fictitious patients (p<0.001) ^37^; TC facts and TSE advice (p=0.002) ^38^; and a TC university campaign (p<0.001) ^35^. Briefing sessions by a physician increased the acceptability of clinical testicular examination but failed to increase men’s willingness to get their testes examined by a clinician ^29^. Moreover, messages written using implementation intentions statements did not significantly increase men’s intentions to perform TSE but significantly increased TSE behaviours ^30^. Other studies that significantly increased TSE behaviours include: shower gel sachets, waterproof stickers, and posters (p=0.006) ^33^; multimodal intervention comprising lectures, discussions, role-plays, posters, pamphlets, booklets, and screening sessions (p<0.001) ^31^; TC and TSE factsheets and testimonies from fictitious patients (p<0.05) ^37^; and a university campaign (p<0.001) ^35^.

In terms of significant TSE reporting, Rovito *et al.* found that 3 out of the 10 reviewed studies did not significantly increase TSE behaviours ^28^. These included: an intervention comparing the effect of print material and shower cards versus video on TSE and shower cards versus no information ^39^; TSE information on shower gel sachets and waterproof stickers and posters versus no information ^33^; and a brochure and checklist to perform TSE versus film with information ^40^.

### Help-seeking behaviours and intentions for testicular symptoms

None of the reviewed experimental studies explored help-seeking for testicular symptoms. In addition, only two of the four cross-sectional studies reviewed by Saab *et al.* ^20^ addressed help-seeking for testicular symptoms ^41, 42^.

### Health behaviours in relation to testicular cancer and self-examination

The reviewed experimental studies addressed men’s health beliefs at pre- and post-test using the five sub-dimensions of the Champion Health Belief Model (i.e. perceived susceptibility, severity, benefits of TSE, barriers to TSE, and self-efficacy) ^26, 27^. Perceived susceptibility, severity, benefits of TSE, and confidence increased (p=0.001) and perceived barriers decreased significantly (p=0.001) 3 months following exposure to a 45-min presentation (Group 1) and pamphlet (Group 2) ^26^. Exposure to TC and TSE education using a PowerPoint presentation, video, pamphlet, and question-answer interaction led to a significant decrease in perceived susceptibility (p=0.001) and an increase in perceived benefits of TSE at 3 months post-test ^27^. By contrast, perceived severity, barriers to TSE, and self-efficacy did not vary significantly ^27^.

## Discussion

### Summary of evidence

A total of five papers were included in the present review. Two were experimental studies and three were structured literature reviews. Overall, the reviewed literature showed that there was an increase in men’s awareness of TC and TSE and behaviours and intentions to perform TSE in response to various interventions. By contrast, help-seeking behaviours and intentions for testicular symptoms were not explored and interventions aimed at raising men’s awareness of BTDs were also lacking.

Examples of interventions that successfully increased men’s awareness of TC and TSE included: a university campaign that involved the use of TC “flyers, brochures, posters, shower cards, bulletin boards, social networking sites, videos, newspaper advertisements, a website, and mass media” (p.305) ^35^; a television show featuring a celebrity with TC ^36^; and TC and TSE factsheets and testimonies from fictitious patients ^37^. By contrast, none of the reviewed interventions aimed to raise men’s awareness of BTDs. Of note, BTDs are more common than TC and a delay in help-seeking for benign testicular symptoms is also linked to negative health outcomes. For instance, a delay of more than 6 hours for pain caused by testicular torsion significantly reduces the chances of salvaging an ischemic testis ^7^. Likewise, untreated epididymitis can lead to severe orchitis, sepsis, and in some cases irreversible infertility ^5, 6^.

The majority of the studies reviewed by Rovito *et al.* ^28^ and Saab *et al.* ^19^ were successful in increasing men’s awareness of TSE and behaviours and intentions to perform TSE. A Cochrane review conducted by Ilic and Misso ^14^ found no definitive evidence regarding the risks and benefits of regular TSE; therefore it was recommended that at-risk groups, such as men with a family history of TC, undescended testis, or testicular atrophy, ought to be advised by their physician regarding the risks (e.g. false positives and concomitant anxiety) and benefits (e.g. early detection) of TSE. As a result, whether to conduct monthly TSE has been polarised into two competing positions. The U.S. Preventive Services Task Force “recommends against screening for testicular cancer in adolescent or adult men” ^15^. Proponents of monthly TSE, however, argue that such recommendations are not based on definitive evidence ^16^. Saab *et al.* called for a middle ground, whereby men are taught how to feel their testes and establish a baseline of what is normal for them without necessarily promoting “scheduled” TSE ^8^.

As stated, help-seeking was not addressed in the reviewed literature. A number of quantitative and qualitative descriptive studies found that men’s intentions to seek help for testicular symptoms (e.g. lumpiness, swelling, and pain) are low ^41– 43^. Saab *et al.* conducted a qualitative descriptive study to explore men’s (n=29) awareness of testicular disorders and intentions to seek help for testicular symptoms ^43^. It was found that a number of men lacked awareness of testicular disorders in general and BTDs in particular, as a result many reported that they would most likely delay help-seeking. In addition to lack of awareness, the following were identified as barriers to help-seeking: lack of familiarity with own testes, symptom misappraisal, low perceived risk of TC, embarrassment, fear, denial, false optimism, fatalism, machoism, stoicism, false reassurance by others, and healthcare system barriers such as access, cost and waiting time ^43^. By contrast, the following were identified as facilitators to help-seeking: personal or family history of a testicular disease, inherent health-seeking drive, and access to support ^43^.

Contradictory evidence in relation to health beliefs (i.e. perceived susceptibility, severity, benefits of TSE, barriers to TSE, and self-efficacy) was found in the reviewed literature. For instance, perceived susceptibility increased following TC and TSE education in the study by Akar and Bebiş ^26^, and decreased following a similar educational approach in the study by Pour *et al.* ^27^. These findings echo findings from studies conducted in different cultural contexts. Muliira *et al.* found that perceived risk of TC was low among Ugandan men ^44^, whereas participants in a study conducted by Rovito *et al.* in the USA scored high on perceived TC vulnerability ^45^. Of note, low perceived TC risk was identified as one of the barriers to seeking help for testicular symptoms ^43^.

None of the reviewed studies reported on whether men’s preferred learning strategies were taken into account during intervention design and delivery. Saab *et al.* interviewed 29 men about their preferred strategies for learning about testicular disorders ^46^. Overall, participants were open to learning about testicular disorders and recommended interventions that are brief, interactive, simple, and light-hearted rather than funny/cheeky ^46^. Thornton warned against the use of “cheeky” humour and puns as these can be potentially offensive and ineffective ^47^. Another factor that should be considered in the design and delivery of health promotion interventions is the literacy and health literacy levels of men. A meta-narrative systematic review of 31 studies exploring men’s information-seeking behaviours in relation to cancer prevention found that younger men and those with high literacy and health literacy levels were more likely to engage with information delivered using technological means. By contrast, men who were older, belonged to ethnic minorities, and had low literacy and health literacy levels were more likely to engage with health information delivered by peers, physicians, and churches ^48^.

### Strengths and limitations

Rigour was ensured by following the guidance of the Cochrane Handbook for Systematic Reviews of Interventions ( http://handbook-5-1.cochrane.org/) and systematically reporting this review using the PRISMA checklist. Moreover, a thorough search of electronic databases, trial registries, grey literature, and reference lists was conducted, and records were independently screened by more than one reviewer to avoid omitting important records. However, the search was limited to records published in English between 2014 and 2018, which increases the risk of study selection bias, and only findings that were relevant to the review outcomes were discussed, which increases the risk of reporting bias. Moreover, the level of evidence per outcome was low, the methodological quality of the reviewed experimental studies was poor, and both experimental studies were not sufficiently powered, which negatively impacts on the assumptions and recommendations from the reviewed studies.

## Conclusions

The present review has implications for research and clinical practice, which should be considered carefully in light of the low level of evidence, relatively poor methodological quality, and small sample sizes. From a research perspective, there is a need for interventions to promote men’s awareness of testicular disorders and to increase their intentions to seek help for testicular symptoms. This could be achieved through considering the information needs and the preferred learning strategies of at-risk age groups, while accounting for sociodemographic variations within these groups ^46^. It is also essential to factor in disorders other than TC, as these were underexplored in the reviewed literature, and to conduct rigorous high-quality studies that capture the impact of the interventions on behaviours longitudinally. Examples include but are not limited to: virtual and augmented reality interventions, gaming technologies, and interactive websites. There is also a need for studies to explore the risks and benefits of TSE, as those were not established in past studies.

The use of theory in intervention design and delivery is key, since interventions with a theoretical underpinning are more likely to achieve the desired outcomes, particularly when there is congruence between the assumptions of the theory and those of the proposed intervention ^49^. An example is the Health Belief Model, which was used in two of the reviewed studies ^26, 27^. Another example is the Preconscious Awareness to Action Framework, a novel theoretical framework developed by Saab *et al.* to raise testicular awareness and promote early help-seeking for testicular symptoms ^8^.

From a practical standpoint, clinicians involved in health promotion are encouraged to direct men to resources where information on testicular disorders is freely and readily accessible. Given the scarcity of high-quality evidence to support scheduled TSE, lack of consensus regarding monthly TSE, clinicians ought to promote testicular awareness by encouraging men to become familiar with the look and feel of their own testes and to seek prompt medical attention for symptoms of testicular disease ^8^.

## Data availability

No data is associated with this article.

## Supplementary material

**Supplementary File 1. Completed PRISMA checklist.**

## References

[1] Manecksha RP Fitzpatrick JM: Epidemiology of testicular cancer. *BJU Int.* 2009;104(9 Pt B):1329–1333. 10.1111/j.1464-410X.2009.08854.x 19840008

[2] Rosen A Jayram G Drazer M: Global trends in testicular cancer incidence and mortality. *Eur Urol.* 2011;60(2):374–379. 10.1016/j.eururo.2011.05.004 21612857

[3] Albers P Albrecht W Algaba F: Guidelines on Testicular Cancer: 2015 Update. *Eur Urol.* 2015;68(6):1054–68. 10.1016/j.eururo.2015.07.044 26297604

[4] Hanna NH Einhorn LH: Testicular cancer--discoveries and updates. *N Engl J Med.* 2014;371(21):2005–2016. 10.1056/NEJMra1407550 25409373

[5] Wampler SM Llanes M: Common scrotal and testicular problems. *Prim Care.* 2010;37(3):613–626. 10.1016/j.pop.2010.04.009 20705202

[6] Srinath H: Acute scrotal pain. *Aust Fam Physician.* 2013;42(11):790–792. 24217099

[7] Bayne CE Villanueva J Davis TD: Factors Associated with Delayed Presentation and Misdiagnosis of Testicular Torsion: A Case-Control Study. *J Pediatr.* 2017;186:200–204. 10.1016/j.jpeds.2017.03.037 28427778

[8] Saab MM Landers M Hegarty J: The Preconscious Awareness to Action Framework: An Application to Promote Testicular Awareness. *Nurs Res.* 2018;67(2):169–176. 10.1097/NNR.0000000000000268 29489637

[9] Rovito MJ Leone JE Cavayero CT: "Off-Label" Usage of Testicular Self-Examination (TSE): Benefits Beyond Cancer Detection. *Am J Mens Health.* 2018;12(13):505–513. 10.1177/1557988315584942 25990509 PMC5987946

[10] Saab MM Landers M Hegarty J: Testicular Cancer Awareness and Screening Practices: A Systematic Review. *Oncol Nurs Forum.* 2016;43(1):E8–E23. 10.1188/16.ONF.E8-E23 26679456

[11] Roy RK Casson K: Attitudes Toward Testicular cancer and Self-Examination Among Northern Irish Males. *Am J Mens Health.* 2017;11(2):253–261. 10.1177/1557988316668131 27645516 PMC5675290

[12] Saleem D Muneer S Younus Khan RF: Knowledge, Attitude and Practices Regarding Benign Testicular Disorders in the Educated Young Men of Pakistan. *Cureus.* 2017;9(8):e1563. 10.7759/cureus.1563 29057175 PMC5640388

[13] Yap LC Keenan R Khan J: Parental awareness of testicular torsion amongst Irish parents. *World J Urol.* 2018;1–4. 10.1007/s00345-018-2269-8 29594530

[14] Ilic D Misso ML: Screening for testicular cancer. *Cochrane Database Syst Rev.* 2011;16(2):CD007853. 10.1002/14651858.CD007853.pub2 21328302

[15] U.S. Preventive Services Task Force: Screening for testicular cancer: U.S. Preventive Services Task Force reaffirmation recommendation statement. *Ann Intern Med.* 2011;154(7):483–486. 10.7326/0003-4819-154-7-201104050-00006 21464350

[16] Rovito MJ Manjelievskaia J Leone JE: From ‘D’ to ‘I’: A critique of the current United States preventive services task force recommendation for testicular cancer screening. *Prev Med Rep.* 2016;3:361–366. 10.1016/j.pmedr.2016.04.006 27419037 PMC4929233

[17] McGuinness LA Obeidat S Hickerton B: Has increasing public health awareness influenced the size of testicular tumours among adult populations over the last 40 years? *J Public Health (Oxf).* 2017;39(1):90–94. 10.1093/pubmed/fdw014 26944075

[18] Aberger M Wilson B Holzbeierlein JM: Testicular self-examination and testicular cancer: a cost-utility analysis. *Cancer Med.* 2014;3(6):1629–1634. 10.1002/cam4.318 25103095 PMC4298389

[19] Saab MM Landers M Hegarty J: Promoting Testicular Cancer Awareness and Screening: A Systematic Review of Interventions. *Cancer Nurs.* 2016;39(6):473–487. 10.1097/NCC.0000000000000333 26859280

[20] Saab MM Landers M Hegarty J: Males’ Awareness of Benign Testicular Disorders: An Integrative Review. *Am J Mens Health.* 2018;12(3):556–566. 10.1177/1557988315626508 26783155 PMC5987954

[21] Garner P Hopewell S Chandler J: When and how to update systematic reviews: consensus and checklist. *BMJ.* 2016;354:i3507. 10.1136/bmj.i3507 27443385 PMC4955793

[22] Moher D Liberati A Tetzlaff J: Preferred reporting items for systematic reviews and meta-analyses: the PRISMA statement. *PLoS Med.* 2009;6(7):1–6, e1000097. 10.1371/journal.pmed.1000097 19621072 PMC2707599

[23] Moyer A Schneider S Knapp-Oliver SK: Published versus unpublished dissertations in psycho-oncology intervention research. *Psychooncology.* 2010;19(3):313–317. 10.1002/pon.1561 19353515 PMC2832099

[24] Guyatt GH Oxman AD Vist GE: GRADE: an emerging consensus on rating quality of evidence and strength of recommendations. *BMJ.* 2008;336:924–926. 10.1136/bmj.39489.470347.AD 18436948 PMC2335261

[25] Shea BJ Reeves BC Wells G: AMSTAR 2: a critical appraisal tool for systematic reviews that include randomised or non-randomised studies of healthcare interventions, or both. *BMJ.* 2017;358:j4008. 10.1136/bmj.j4008 28935701 PMC5833365

[26] Akar ŞZ Bebiş H: Evaluation of the effectiveness of testicular cancer and testicular self-examination training for patient care personnel: intervention study. *Health Educ Res.* 2014;29(6):966–976. 10.1093/her/cyu055 25248831

[27] Pour HA Kunter D Norouzzadeh R: The Effect of Testicular Self-Examination Education on Knowledge, Performance, and Health Beliefs of Turkish Men. *J Canc Educ.* 2018;33(2):398–403. 10.1007/s13187-016-1132-0 27815814

[28] Rovito MJ Cavayero C Leone JE: Interventions Promoting Testicular Self-Examination (TSE) Performance: A Systematic Review. *Am J Mens Health.* 2015;9(6):506–518. 10.1177/1557988314555360 25359870

[29] Kedzierewicz R Chargari C Le Moulec S: Knowledge and screening of testicular cancer in the French armed forces: a prospective study. *Mil Med.* 2011;176(10):1188–1192. 10.7205/MILMED-D-11-00037 22128657

[30] Steadman L Quine L: Encouraging young males to perform testicular self-examination: a simple, but effective, implementation intentions intervention. *Br J Health Psychol.* 2004;9(Pt 4):479–487. 10.1348/1359107042304551 15509356

[31] Shallwani K Ramji R Ali TS: Self-examination for breast and testicular cancers: a community-based intervention study. *Asian Pac J Cancer Prev.* 2010;11(1):145–148. 20593946

[32] Folkins A Sadler GR Ko C: Improving the deaf community's access to prostate and testicular cancer information: a survey study. *BMC Public Health.* 2005;5:63. 10.1186/1471-2458-5-63 15938751 PMC1180455

[33] McCullagh J Lewis G Warlow C: Promoting awareness and practice of testicular self-examination. *Nurs Stand.* 2005;19(51):41–49. 10.7748/ns2005.08.19.51.41.c3944 16161515

[34] Sacks L Nakaji M Harry KM: Testicular cancer knowledge among deaf and hearing men. *J Cancer Educ.* 2013;28(3):503–508. 10.1007/s13187-013-0493-x 23813488 PMC3770485

[35] Wanzer MB Foster SC Servoss T: Educating young men about testicular cancer: support for a comprehensive testicular cancer campaign. *J Health Commun.* 2014;19(3):303–320. 10.1080/10810730.2013.811320 24117344

[36] Trumbo CW: Mass-mediated information effects on testicular self-examination among college students. *J Am Coll Health.* 2004;52(6):257–61. 10.3200/JACH.52.6.257-262 15134099

[37] Umeh K Chadwick R: Early detection of testicular cancer: revisiting the role of self-efficacy in testicular self-examination among young asymptomatic males. *J Behav Med.* 2016;39(1):151–160. 10.1007/s10865-010-9262-z 20411318

[38] Evans RE Beeken RJ Steptoe A: Cancer information and anxiety: applying the Extended Parallel Process Model. *J Health Psychol.* 2012;17(4):579–589. 10.1177/1359105311421046 21914768

[39] Brown CG Patrician PA Brosch LR: Increasing testicular self-examination in active duty soldiers: an intervention study. *Medsurg Nurs.* 2012;21(2):97–102. 22667002

[40] Finney JW Weist MD Friman PC: Evaluation of two health education strategies for testicular self-examination. *J Appl Behav Anal.* 1995;28(1):39–46. 10.1901/jaba.1995.28-39 7706149 PMC1279784

[41] Nasrallah P Nair G Congeni J: Testicular health awareness in pubertal males. *J Urol.* 2000;164(3 Pt 2):1115–1117. 10.1016/S0022-5347(05)67265-5 10958755

[42] Congeni J Miller SF Bennett CL: Awareness of genital health in young male athletes. *Clin J Sport Med.* 2005;15(1):22–26. 10.1097/00042752-200501000-00005 15654187

[43] Saab MM Landers M Hegarty J: Exploring awareness and help-seeking intentions for testicular symptoms among heterosexual, gay, and bisexual men in Ireland: A qualitative descriptive study. *Int J Nurs Stud.* 2017;67:41–50. 10.1016/j.ijnurstu.2016.11.016 27915088

[44] Muliira JK Nalwanga PB Muliira RS: Knowledge, perceived risk and barriers to testicular self-examination among male university students in Uganda. *J Mens Health.* 2013;9(1):36–44. 10.1016/j.jomh.2011.11.004

[45] Rovito MJ Gordon TF Bauerle SB: Perceptions of testicular cancer and testicular self-examination among college men: a report on intention, vulnerability, and promotional material preferences. *Am J Mens Health.* 2011;5(6):500–507. 10.1177/1557988311409023 21659352

[46] Saab MM Landers M Hegarty J: Exploring men's preferred strategies for learning about testicular disorders inclusive of testicular cancer: A qualitative descriptive study. *Eur J Oncol Nurs.* 2017;26:27–35. 10.1016/j.ejon.2016.11.001 28069149

[47] Thornton CP: Best Practice in Teaching Male Adolescents and Young Men to Perform Testicular Self-Examinations: A Review. *J Pediatr Health Care.* 2016;30(6):518–527. 10.1016/j.pedhc.2015.11.009 26778347

[48] Saab MM Reidy M Hegarty J: Men's information-seeking behavior regarding cancer risk and screening: A meta-narrative systematic review. *Psychooncology.* 2018;27(2):410–419. 10.1002/pon.4506 28728212

[49] Michie S Johnston M Francis J: From theory to intervention: Mapping theoretically derived behavioural determinants to behaviour change techniques. *Appl Psychol.* 2008;57(4):660–680. 10.1111/j.1464-0597.2008.00341.x

