# Promoting men’s awareness, self-examination, and help-seeking for testicular disorders: a systematic review of interventions

**DOI:** 10.12688/hrbopenres.12837.3

**Published:** 2023-10-13

**Authors:** Mohamad M. Saab, Martin P. Davoren, Aileen Murphy, David Murphy, Eoghan Cooke, Margaret Landers, Serena Fitzgerald, Noel Richardson, Michael J. Rovito, Christian Von Wagner, Mike Murphy, Darren Dahly, Josephine Hegarty

**Affiliations:** 1 School of Nursing and Midwifery, University College Cork, Cork, Ireland; 2 School of Public Health, University College Cork, Cork, Ireland; 3 Sexual Health Centre, Cork, Ireland; 4 Department of Economics, University College Cork, Cork, Ireland; 5 School of Computer Science & Information Technology, University College Cork, Cork, Ireland; 6 Health Research Board Clinical Research Facility, University College Cork, Cork, Ireland; 7 Health Research Board National Clinical Trials Office, College of Medicine & Health, University College Cork, Cork, Ireland; 8 Department of Science and Health, South East Technological University, Carlow, Ireland; 9 College of Health Professions and Sciences, University of Central Florida, Orlando, Florida, USA; 10 Behavioural Science and Health, Institute of Epidemiology & Health, University College London, London, UK; 11 School of Applied Psychology, University College Cork, Cork, Ireland

**Keywords:** Awareness, health promotion, help-seeking, men’s health, systematic review, testicular cancer, testicular diseases, testicular self-examination

## Abstract

**Background:** Testicular cancer (TC) is among the most commonly diagnosed cancers in men aged 15–40 years. The incidence of TC is on the rise. Benign testicular disorders, such as testicular torsion and epididymitis, can lead to testicular ischemia, sepsis, and infertility if left untreated. This updated systematic review aims to evaluate the effectiveness of studies promoting men’s knowledge and awareness of testicular disorders and/or self-examination, behaviours and/or intentions to examine their testes, and help-seeking behaviours and/or intentions for testicular disorder symptoms.

**Methods:** Academic Search Complete, Medline, CINAHL, PsycINFO, ERIC, the Cochrane Library, the World Health Organisation International Clinical Trials Registry Platform, and Clinicaltrials.gov were searched for studies published between April 2018 and August 2023. Methodological quality was assessed and results were synthesised meta-narratively.

**Results:** Five studies were included. The majority of the reviewed interventions were successful in increasing men’s awareness of TC and self-examination, including a PowerPoint presentation, an online educational brochure, video-assisted teaching, a motivational video, and a virtual reality game. Only one study addressed help-seeking for testicular symptoms and promoted men’s awareness of benign as well as malignant testicular diseases.

**Conclusions:** This review highlights the importance of evaluating innovative educational interventions aimed at younger men, whilst raising their awareness of testicular disorders and increasing their help-seeking intentions for testicular disorder symptoms. Given the lack of consensus around scheduled testicular self-examination among younger men, clinicians are encouraged to instruct men to familiarise themselves with the look and feel of their own testes and to seek timely medical attention for abnormalities.

**Registration:** The protocol of the previous version of this review was registered with the International Prospective Register of Systematic Reviews (PROSPERO) under the registration number CRD42018093671.

## Introduction

According to the National Cancer Institute, testicular cancer (TC) is among the most commonly diagnosed cancers in men aged 15 to 40 years. The incidence of TC has doubled globally over the past 40 years and is highest in Western and Northern European countries, Australia, and North America ^1, 2^. According to the National Cancer Registry Ireland, over 90% of TC cases and 85% of TC deaths in Ireland occur among men younger than 50 years. Furthermore, the incidence of TC in Ireland is increasing by 2.4% annually. A unilateral painless testicular mass is a classical sign of TC. Testicular pain, back pain, cough, haemoptysis, and headaches can be warning signs of metastatic TC ^3, 4^.

Benign testicular disorders (BTDs) can also have a negative impact on a man’s health. Epididymo-orchitis, often contracted sexually by men younger than 50 years, is known to be the primary cause of acute scrotal pain and testicular enlargement. This infection can cause sepsis and infertility if not diagnosed and managed promptly ^5^. Testicular torsion is characterised by severe scrotal pain, oedema, nausea, and vomiting, and can lead to testicular ischemia and necrosis if testicular perfusion is not restored within 6 hours from the onset of pain ^5– 7^. The severity of these conditions highlights the potential role of testicular awareness and testicular self-examination (TSE) in detecting TC as well as BTDs ^8, 9^.

A systematic review of 25 studies exploring men’s awareness of TC and TSE found that men were unaware of TC risk factors, signs and symptoms, and treatments, and that very few reported performing TSE ^10^. These findings were echoed by Roy and Casson, who explored the awareness, knowledge, and attitudes regarding TC and TSE of 150 men in Northern Ireland ^11^. This study found that only 39% of participants correctly identified the TC at-risk age group, and only 17% were aware of TSE ^11^.

Sparse recent evidence exists in relation to BTD awareness. Saleem *et al.* explored men’s awareness of BTDs in Pakistan and found that 78.8% of participants were unaware of the symptoms of BTDs, 73.6% reported that BTDs were considered taboo, and 29.8% did not intend to perform TSE ^12^. Yap *et al.* surveyed Irish parents (n=242) about their awareness and help-seeking for testicular torsion ^13^. This study found that parents who were aware of torsion were four times more likely to seek immediate help (OR, 4.2; 95% CI, 1.4-12.2; p<0.01) than those who lacked awareness. Moreover, participants who correctly identified the timeframe for help-seeking were three times more likely to seek immediate help than those who did not know the timeframe (OR, 3.0; 95% CI, 0.85-10.8; p=0.08) ^13^.

There is no consensus regarding the effectiveness of monthly TSE in detecting testicular disorders early ^14^, which resulted in different recommendations regarding this practice globally. For instance, the U.S. Preventive Services Task Force opposes this practice ^15^, whereas Cancer Research UK and the Irish Cancer Society encourage men to check their testes and report any abnormalities to a healthcare professional. TSE proponents were critical of the decision made by U.S. Preventive Services Task Force and stated that TSE has potential benefits beyond the early detection of TC such as familiarising men with their own testes and helping detect TC and BTDs early ^16^. McGuinness *et al.* highlighted that public health initiatives promoting TSE were linked to early TC diagnosis and smaller tumour size at diagnosis ^17^. Furthermore, in their cost-utility analysis of TC and TSE, Aberger *et al.* found that a 2.4 to 1 cost-benefit ratio was established for early-onset versus advanced TC ^18^, which emphasises the importance of raising men’s awareness of diseases of the testes.

Saab *et al.* systematically reviewed evidence from 11 experimental studies (2004–2014) promoting men’s awareness of TC and TSE, and increasing their TSE intentions and behaviours ^19^. Saab *et al.* also conducted an integrative review of the literature on BTD awareness (1985–2015) ^20^. Despite men’s lack of awareness of BTDs and their intentions to delay help-seeking for symptoms of testicular disease, none of these reviews included studies that aimed at promoting men’s awareness of BTDs and/or increasing their intentions to seek help for testicular symptoms. The present review builds upon the search, screening, and output from both reviews ^19, 20^. Of note, there is no gold standard for the frequency of updating structured reviews ^21^. However, biennial review updates are recommended by the Cochrane Library.

### Objectives

The aim of this updated systematic review is to evaluate the effectiveness of experimental studies promoting men’s knowledge and awareness of testicular disorders and/or self-examination, behaviours and/or intentions to examine their testes, and help-seeking behaviours and/or intentions for testicular symptoms. The outcomes of this review are presented below using the PICOS (participants, interventions, comparisons, outcomes, and study design) framework:

Outcomes:

1. The effect of intervention on men’s knowledge and awareness of testicular disorders and/or self-examination, compared to baseline and/or control conditions (i.e., alternative intervention or no intervention).

2. The effect of intervention on men’s behaviours and/or intentions to examine their testes, compared to baseline and/or control conditions (i.e., alternative intervention or no intervention).

3. The effect of intervention on men’s help-seeking behaviours and/or intentions for testicular disorder symptoms.

## Methods

### Protocol and registration

This updated systematic review is reported using the Preferred Reporting Items for Systematic Reviews and Meta-Analysis (PRISMA) checklist ^22^. The review questions and methods were predetermined and were not amended during the review process. The protocol of the original review was registered with the International Prospective Register of Systematic Reviews (PROSPERO) under the registration number CRD42018093671.

### Eligibility criteria

Studies were eligible for inclusion if they used any experimental design and were conducted among men who did not have a diagnosis of a testicular disorder. Studies addressing the review outcomes and studies evaluating the effect of intervention(s) compared to baseline and/or control conditions were included. The full inclusion criteria are reported in Table 1 using the PICOS framework.

**Table 1.** Review inclusion criteria using the PICOS framework.

**Participants**	Adult men (aged 18+) without a diagnosis of a testicular disorder
**Interventions **	Educational/health promotion intervention/programme
**Comparisons **	The effect of intervention compared to baseline and/or control conditions i.e., alternative intervention(s) or no intervention
**Outcomes**	(i) Knowledge and awareness of testicular disorders and/or self-examination (ii) Behaviours and/or intentions to examine/feel own testes (iii) Help-seeking behaviours and/or intentions for testicular disorder symptoms
**Study design **	Any experimental design (i.e., randomised controlled trial, non-randomised controlled trial, pre-post study design with one or more groups, and post-test only study design with one or more groups)

Men with a diagnosis of a testicular disorder, studies with women only, studies with a paediatric population, and studies where findings from men, women, and/or paediatric populations are indistinguishable were excluded. Additionally, quantitative descriptive studies, qualitative studies, opinion papers, reviews of the literature, and conference abstracts were not eligible for inclusion. Theses and dissertations were also excluded because the merit of their use in systematic reviews is questionable ^23^.

### Information sources and search strategy

The following electronic databases were searched in August 2023: Academic Search Complete, Medline, CINAHL, PsycINFO, ERIC, and The Cochrane Library. In addition, eligible studies were sought from trial registries including the World Health Organisation International Clinical Trials Registry Platform (ICTRP) and Clinicaltrials.gov. Reference lists of eligible papers were also reviewed. As this is an updated version of a previous review, the search was limited to records published between April 2018 and August 2023.

The following keywords were searched based on title and abstract using Boolean operators “OR” and “AND”: “testicular disease*” OR “testicular disorder*” OR “testicular cancer” OR “testicular neoplas*” OR “testicular tumor*” OR “testicular tumour*” OR “testicular malignan*” OR “benign testicular disorder*” OR “benign testicular disease*” OR “testicular torsion” OR epididymitis OR orchitis OR epididymo-orchitis OR hydrocele OR varicocele OR spermatocele OR “testicular symptom*” OR “testicular pain” OR “testicular lump*” OR “testicular swelling” OR “scrot* symptom*” OR “scrot* pain” OR “scrot* lump*” OR “scrot* swelling” AND knowledge OR awareness OR practice* OR self-exam* OR “self exam*” OR feel* OR screen* OR “early detect*” OR help-seeking OR “help seeking” OR “help-seeking intention*” OR “help seeking intention*” OR “help-seeking behavior*” OR “help-seeking behaviour*” OR “help seeking behavior” OR “help seeking behaviour” AND intervention* OR inform* OR educat* OR “health education” OR “health promotion” OR trial* OR experiment* OR stud* OR program*.

### Study selection and data extraction

Records identified from electronic databases and trial registries were exported to Covidence, an online software used to facilitate screening and data extraction. Duplicates were deleted automatically in Covidence.

All records were screened based on title and abstract. Following the exclusion of irrelevant records, the full text of potentially eligible studies was obtained for further screening. Title, abstract, and full-text screenings were conducted by two independent reviewers. Screening conflicts were resolved by consensus.

A standardised extraction table was used to extract data from experimental studies ^19, 20^. Data were extracted by one reviewer and cross-checked for accuracy by a second reviewer. The following data were extracted: author(s) and year; aim(s); country, setting and funding; participants; design and theoretical underpinning; intervention(s); outcome(s) and data collection; and findings presented according to the review questions.

### Quality assessment

The methodological quality of the included studies was appraised using the Mixed Method Appraisal Tool (MMAT) which allows the appraisal of various study designs ^24^. In the context of the present review, the quality of randomised controlled trials (RCT) and non-RCTs was appraised. Voting on each quality item was conducted on a “yes”, “no”, and “cannot tell” basis. Quality appraisal was conducted by one author and verified by a second author.

### Data synthesis

A meta-analysis with summary measures of treatment effect using weighted/standard mean difference, risk/odds ratios, and 95% confidence was planned using RevMan 5, if the included studies were sufficiently homogenous. However, the included studies were heterogeneous in terms of intervention format, data collection, study design, and participant allocation; therefore, findings from the reviewed studies were synthesised meta-narratively.

## Results

### Study selection

**Figure 1.**
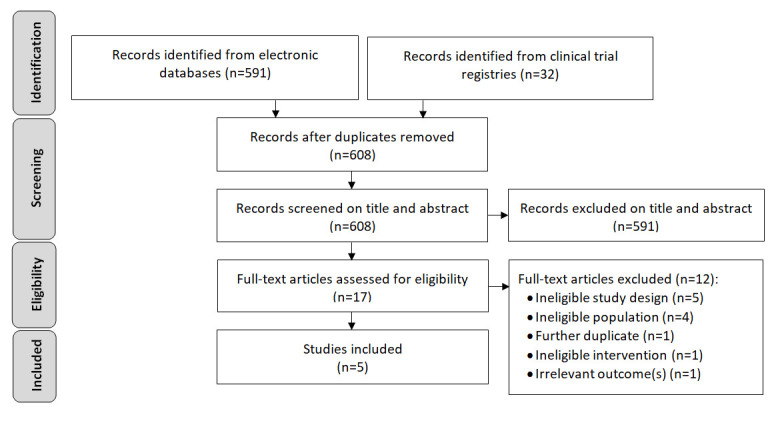
Flow diagram detailing study identification, screening, and selection process.

A total of 623 records were identified from electronic databases and clinical trial registries. No additional records were identified from reference list checks. Following the exclusion of duplicates, 608 records were screened based on title and abstract. Of those, 17 full-text articles were assessed for eligibility and 12 were excluded. Five studies were included in the present review. The full study selection process and reasons for exclusion are presented in Figure 1.

### Study characteristics

Two studies were conducted in Turkey ^25, 26^. The remaining studies were conducted in Ireland ^27^, India ^28^, and Pakistan ^29^. Three of the studies had a theoretical underpinning including the Health Belief Model ^25, 26^ and the Pre-Conscious Awareness to Action Framework ^27^. Two studies explored awareness of TC and TSE and TSE behaviours ^25, 29^. Sagir and Alitnel ^26^ addressed TSE behaviours only, Shenbagagap *et al.* ^28^ explored TSE awareness only, while Saab *et al.* ^27^ explored TC and TSE awareness, TSE intentions and help-seeking behaviours for testicular symptoms. Sample sizes ranged from 49 ^27^ to 124 ^29^ participants. Two studies used a quasi-experimental design ^26, 28^, two were pre-post pilot studies ^27, 29^, and the remaining study was an RCT ^25^.

### Quality assessment

All five studies had clear research questions and used appropriate data collection methods. The RCT met all the MMAT criteria ^25^. As for non-RCTs (n=4), only two studies accounted for confounders and reported that participants were representative of the target population ^26, 27^. It was unclear in two studies if measurements were appropriate regarding both, outcome and intervention ^28, 29^. While it was only unclear in one study if the intervention was administered as intended ^28^. The four non-RCTs met the remaining MMAT criteria ( Table 2).

**Table 2.** Quality assessment using the mixed method appraisal tool.

Study designs	Author(s) & year	Quality appraisal items *											
		1	2	3	4	5	6	7	8	9	10	11	12
Non-randomised studies **	Saab *et al.* (2018)	Y	Y	Y	Y	Y	Y	Y					
	Sagir and Altinel (2023)	Y	Y	Y	Y	Y	Y	Y					
	Shenbagapraba *et al.* (2020)	Y	Y	CT	CT	Y	CT	CT					
	Waheed *et al.* (2023)	Y	Y	CT	CT	Y	CT	Y					
Randomised controlled studies ***	Akcali and Tastan (2023)	Y	Y						Y	Y	Y	Y	Y

*All studies: 1=Clear research questions/aims 2=Data collected address research question/aims **Non-randomised studies: 3=Participants representative of target population 4=Measurements appropriate regarding both the outcome and the intervention 5=Complete outcome data 6=Confounders accounted for in the design and analysis 7=The intervention administered as intended ***Randomised controlled studies: 8=Randomisation appropriately performed 9=Groups comparable at baseline 10=There are complete outcome data 11=Outcome assessors blinded to the intervention 12=Participants adhered to the assigned intervention **Abbreviations:** CT=Can’t Tell; N=No; Y=Yes.

### Synthesis of results

The full data extraction table and findings from individual studies are presented in Table 3.

**Table 3.** Data extraction and summary of findings from individual studies (n=5).

Author(s) & year	Aim(s)	Country, setting & funding	Participants	Design & theoretical underpinning	Intervention(s)	Outcome(s) and data collection	Findings *
Akcali and Tastan (2023)	“To examine the effects of the flipped classroom method on knowledge, behaviour and health beliefs on TC and TSE in male nursing students” (p. 231)	Northern Cyprus, Turkey University Funding not reported (NR)	n=66 male nursing students randomly assigned to two groups, *Intervention* * group* (n=34; lecture using flipped classroom model [FCM] group) and *Control * *group* (n=32; traditional lecture group)	Randomized, controlled trial Health Belief Model	*Intervention* * group*: PowerPoint presentation and video about the structure of the testicles, the definition of TC, its incidence, TC risk factors, TC symptoms, diagnostic methods, the importance of TSE and how to do it followed by a 40-min training session designed according to FCM including a Q&A session. *Control group* 30 min traditional PowerPoint presentation	Data collected at pre-test and post-test (6 weeks) using an 11- item descriptive information form, a 16-item knowledge questionnaire on TC and TSE, and 26-item Champion Health Belief Model Scale (CHBMS) with 5 sub-dimensions: perceived: Susceptibility (5) Severity (7) Benefits of TSE (3) Barriers to TSE (5) Self-efficacy (6).	**(O1)** At pre-test, 70.6% (n=24) in the intervention group and 56.3% (n=18) in the control group had no previous knowledge of TC (p>0.05). At post-test, mean knowledge scores were higher in the intervention group (14.44±1.84) vs the control group (12.65±3.89) (p<0.05). **(O2)** At pre-test, 97.1% (n=33) in the intervention group and 90.6% (n=29) in the control group reported not practicing TSE (p> 0.05). At post-test, 82.4% (n=28) in the intervention group reported practicing TSE vs 59.4% (n=19) in the control group (p<0.05). **(O3)** Not reported (NR)
Saab *et al.* (2018)	“To enhance men’s awareness of testicular disorders, help-seeking intentions for testicular symptoms, and intention and behavior to feel their testes” (p. 349)	Ireland University PhD scholarship	n=49 men aged between 18–50 years	Pre-post pilot study Preconscious Awareness to Action Framework	“E-MAT”: A brief three-level virtual reality (VR) experience delivered using a VR headset, a controller with haptic (i.e., vibrational) feedback, and over-ear headphones with voiceover, which provided feedback and information and helped transition between the levels.	Data collected at pretest (T0), immediately post-test (T1), and 1 month post- test (T2) using a sociodemographic questionnaire, a 12-item knowledge questionnaire, 5-item testicular awareness scale, perceived risk item (1-item), implementation intentions scale (3 items), general help seeking questionnaire (3 items) and behaviour questionnaire (3 items)	**(O1)** Mean knowledge scores were 6.2 (±1.8) at T0 vs 9.8 (±1.5) at T1 vs 8.9 (SD±1.8) at T2. T0 vs T1: p<0.001 T2 vs T0: p<0.001 T2 vs T1: p=0.014 Mean testicular awareness scores were 3.6 (SD= 0.6) at T0 vs 3.8 (SD= 0.8) at T1 vs 4 (SD= 0.6) at T2. T0 vs T1: p=.038 T2 vs T0: p<.001 T2 vs T1: p=.033 **(O2)** 40.8% (n=20) reported examining their testes within the past month (T0). 81.6% reported examining their testes at T2 (p <0 .001). At T0, 77.6% (n=38) agreed that they “intend” to feel their testes. At T2, out of the 22.4% (n=11) who did not agree, 54.5% (n=6) reported feeling their testes (p=0.019). At T0, 34.7% (n=17) intended to advise at least one man about the “importance of feeling his own testes”, At T2, out of the 65.3% (n=32) who were not in agreement, 25% (n=8) reported “having advised at least one man to feel his own testes” (p<0.001) **(O3)** 42.9% (n=21) plan to seek information about testicular disorders General help seeking intention scores for swelling were 3.5 (±0.9) at T0 vs 3.8 (±1) at T1 vs 3.9 (±1) at T2. T0 vs T1: p<0.001 T2 vs T0: p=0.01 T2 vs T1: p=1 General help seeking intention scores for lumps were 3.5 (±0.9) at T0 vs 3.8 (±1.1) at T1 vs 3.9 (±1) at T2. T0 vs T1: p=0.003 T2 vs T0: p=0.04 T2 vs T1: p=1 General help seeking intention scores for pain were 3.2 (±0.9) at T0 vs 3.8 (±1.3) at T1 vs 3.7 (±1) at T2. T0 vs T1: p<0.001 T2 vs T0: p<0.001 T2 vs T1: p=1
Sagir and Altinel (2023)	“To examine the effect of an educational brochure about TC and its early diagnosis on the health beliefs and self- examination of participants” (p. 632).	Turkey University Selcuk University Scientific Research Coordination Office	n= 92 students (experimental group: n=48; control group: n=44)	Quasi- experimental study Health belief Model	An online educational brochure about TC and self- examination	Data collected at pre-test and post-test included a personal information form and health beliefs scale for TC and TSE	**(O1)** NR **(O2)** Pre-test, TSE rate was 4.2% in the experiment group vs 2.3% in the control group. Post-test TSE rate was 83.3% in the experimental group and 4.5% of in the control group (p<0.001). **(O3)** NR
Shenbagapraba *et al.* (2020)	“To assess the pre-test knowledge on TSE among men, to assess the effect of video assisted teaching on TSE among men, to associate between pre- test, post-test knowledge regarding TSE among men” (p. 577)	India College No funding	n=50 adult men in college (18–35 years)	Quasi- experimental design No theoretical underpinning	Video assisted teaching intervention	Data collected using a self-structured questionnaire.	**(Q1)** At pre-test, 4% had adequate knowledge, 80% had moderate knowledge, and 16% had inadequate knowledge regarding TSE. At post-test, 74% had adequate knowledge, 26% had moderately adequate knowledge, and none had inadequate knowledge regarding TSE. Knowledge of the Effectiveness of Video Assisted Teaching on TSE mean score was 4.33 pre-test vs 8.47 post-test (p<0.05). **(O2)** NR **(O3)** NR
Waheed *et al.* (2023)	“To acquire the frame of mind regarding TC and TSE among the male outdoor patients of Lahore General Hospital” (p. 1)	Pakistan Hospital Funding NR	n=124 male patients	Pre-post pilot study Theoretical underpinning: NR	88-second bilingual, motivational video on “how to examine your testicles?” and an educational awareness-based pamphlet on TC and TSE	Data was collection at pre-test using a bilingual questionnaire via Google Docs to assess: (i) awareness of TSE (ii) awareness of TC (iii) thoughts and myths about TC (iv) quality of knowledge and satisfaction, and (v) intention of TSE. Data was collected post- test via survey to gather information about participants understanding, satisfaction with the quality of survey, and willingness to teach others about the subject matter.	**(Q1) Pre-test**: 82% had never heard of TC, 93% did not know if TC was most commonly seen in 15–35 aged male groups, 92% did not know if the most important risk factor for TC is ones with undescended testes, 56% did not know if chance of recovery increases by 80–90% with early diagnosis, 65% did not know if the earliest diagnostic method for TC is self- examination, 72% did not know if TC can be prevented as a palpable lump, swollen testes, or heaviness in the testes, 92% had not heard of TSE, 74% agreed that TSE can be vital to detect testicular diseases at an early stage, 65% did not know if TSE should be done in the shower or shortly after the shower, 69% did not know if TSE should be done regularly every month. **Post-test**: 100% claimed that their knowledge of TC improved and 97% were ready to teach other males, 97% agreed to share the knowledge with their male members of family and friends. **(O2) Pre-test**: 92% had not performed TSE, 58.3% mentioned lack of education as the reason for not doing TSE. Of the 8% who performed TSE, 45.5% did it a few times in the last year, while 45.4% did it a few times in the last 6 months. 9.1% performed TSE once a month, 97% never requested to get information regarding TSE, 96% never requested to get information regarding TC. **Post-test**: 76% agreed to do TSE as soon as possible, and 20% within this month, while 4% denied doing TSE. **(O3)** NR

* Findings organised according to the review outcomes as follows: **(O1)** Knowledge and awareness of testicular disorders and/or self-examination **(O2)** Behaviours and/or intentions to examine/feel their testes **(O3)** Help-seeking behaviours and/or intentions for testicular symptoms. **Abbreviations:** CHBM=Champion’s Health Belief Model; CHBMS=Champion Health Belief Model Scale; E-MAT=Enhancing Men’s Awareness of Testicular Diseases; FCM=Flipped Classroom Model; HBM=Health Belief Model; NR=Not Reported; Q&A=Questions and Answers; T0=Time 0 (baseline); T1:Time 1; T2=Time 2; TC=Testicular Cancer; TSE=Testicular Self-Examination; VR=Virtual Reality.

### Awareness of testicular disorders and self-examination

Four of the reviewed studies addressed men’s awareness of TC and TSE ^25, 27– 29^. Akcali and Tastan ^25^ conducted an RCT comparing the effect of two interventions (PowerPoint presentation and video using the Health Belief Model [Group 1] and traditional PowerPoint presentation [Group 2]) on men’s awareness of TC and TSE. The study also assessed men’s health beliefs in relation to TC and TSE ^25^. At pre-test, 70.6% (n=24) in the intervention group and 56.3% (n=18) in the control group had no previous knowledge of TC. Knowledge increased significantly at post-test for both groups but was significantly higher in Group 1 (p<0.05). Saab *et al.* conducted a pre-post study to enhance men’s (n=49) awareness of testicular diseases using a brief virtual reality game ^27^. Testicular knowledge and testicular awareness increased significantly immediately post-test (p<0.05) ^27^. This increase was maintained one month post-test (p=0.033). Waheed *et al*. also conducted a pre-post pilot study to assess men’s (n=124) knowledge regarding TC and TSE ^29^. At pre-test, most participants had never heard of TC (82%, n=102) and TSE (92%, n=94). While almost all participants (93%, n=95) did not know if TC was most commonly seen in 15–35 ages male groups. Likewise, 92% (n=94) did not know that having undescended testes increase the risk of TC. All participants reported that their knowledge of TC improved at post-test and 97% (n=99) agreed to share their knowledge with members of their family and friends ^29^. Shenbagapraba *et al.* used a quasi-experimental design to assess the effect of video assisted teaching on men’s (n=50) knowledge of TSE ^28^. At pre-test, only 4% (n=2) had adequate knowledge regarding TSE. However, 74% (n=37) had adequate knowledge about TSE at post-test ^28^.

### Behaviours and intentions to perform testicular self-examination

TSE behaviours and/or intentions were explored in four of the reviewed papers ^25– 27, 29^. In the study by Akcali and Tastan, only one participant in the intervention group and three in the control group reported practicing TSE ^25^. This increased significantly to 82.4% (n=28) among the intervention group and 59.4% (n=19) among the control group at post-test (p<0.05) ^25^. Over three quarters of participants (77.6%, n=38) in the study by Saab *et al.* reported that they intend to feel their testes at pre-test ^27^. Of the 22.4% (n=11) participants who did not intent to feel their testes, 54.5% (n=6) reported feeling their testes at post-test ^27^. TSE rate in the Sagir and Altinel’s study was 4.2% in the intervention group and 2.3% in the control group at pre-test ^26^. At post-test, this increased significantly to 83.3% in the intervention group and 4.5% in the control group performing a TSE (p<0.001) ^26^. In the study by Waheed *et al.*, 92% of participants had never performed TSE at pre-test with more than half of participants (58.3%) mentioning lack of education as a reason for not doing ^29^. In addition, 97% never requested to get information about TSE, while 96% never asked for information about TC at pre-test. At post-test, 76% agreed to do a TSE as soon as possible, and 20% within that month, while 4% patients denied doing a TSE ^29^.

### Help-seeking behaviours and intentions for testicular symptoms

Only one study addressed men’s help-seeking for testicular symptoms ^27^. Help-seeking intention scores for testicular swelling and testicular lumps were 3.5/7 at pre-test which significantly increased to 3.8/7 immediately post-test for both symptoms (p<0.001 and p=0.003 respectively). Similarly, help-seeking intention scores for testicular pain were 3.2/7 at pre-test which increased significantly to 3.8/7 immediately post-test (p<0.001) ^27^.

## Discussion

### Summary of evidence

Five studies were included in this updated systematic review. Overall, the reviewed literature showed that there was an increase in men’s awareness of TC and TSE and behaviours and intentions to perform TSE in response to various interventions, at least in the short-term. The included studies seldom addressed help-seeking behaviours and intentions for testicular symptoms. Indeed, only one study addressed this outcome and found a significant increase in intentions to seek help for symptoms of concern following a virtual reality game ^27^.

Examples of interventions that successfully increased men’s awareness of TC and TSE included: PowerPoint presentation underpinned by the Health Belief Model ^25^, an online educational brochure also underpinned by the Health Belief Model ^26^, video-assisted teaching ^28^, a motivational video ^29^, and a virtual reality game ^27^. Of note, only Saab *et al.*’s study aimed to promote men’s awareness of BTDs as opposed to only TC ^27^. BTDs are more common than TC and a delay in help-seeking for benign testicular symptoms is also linked to negative health outcomes. For instance, a delay of more than 6 hours for pain caused by testicular torsion significantly reduces the chances of salvaging an ischemic testis ^7^. Likewise, untreated epididymitis can lead to severe orchitis, sepsis, and in some cases irreversible infertility ^5, 6^.

As for TSE, a Cochrane review conducted by Ilic and Misso ^14^ found no definitive evidence regarding the risks and benefits of regular TSE; therefore it was recommended that at-risk groups, such as men with a family history of TC, undescended testis, or testicular atrophy, ought to be advised by their physician regarding the risks (e.g. false positives and concomitant anxiety) and benefits (e.g. early detection) of TSE. As a result, whether to conduct monthly TSE has been polarised into two competing positions. Since 2011, the U.S. Preventive Services Task Force “recommends against screening for testicular cancer in adolescent or adult men” ^15^ (p. 483). Proponents of monthly TSE, however, argue that such recommendations are not based on definitive evidence ^16^. Saab *et al.* called for a middle ground, using the concept “testicular awareness” whereby men are taught how to feel their testes and establish a baseline of what is normal for them without necessarily promoting “scheduled” TSE ^8, 30^.

As stated, help-seeking was only addressed in one study ^27^. A number of quantitative and qualitative descriptive studies found that men’s intentions to seek help for testicular symptoms (e.g., lumpiness, swelling, and pain) are low ^31– 33^. Saab *et al.* conducted a qualitative descriptive study to explore men’s (n=29) awareness of testicular disorders and intentions to seek help for testicular symptoms ^33^. It was found that men lacked awareness of testicular disorders in general and BTDs in particular, as a result many reported that they would most likely delay help-seeking. In addition to lack of awareness, the following were identified as barriers to help-seeking: lack of familiarity with own testes, symptom misappraisal, low perceived risk of TC, embarrassment, fear, denial, false optimism, fatalism, machoism, stoicism, false reassurance by others, and healthcare system barriers such as access, cost and waiting time ^33^. By contrast, the following were identified as facilitators to help-seeking: personal or family history of a testicular disease, inherent health-seeking drive, and access to support ^33^.

Only Saab *et al.* considered men’s preferred learning strategies during intervention design and delivery ^27^. Previously, Saab *et al.* interviewed 29 men about their preferred strategies for learning about testicular disorders ^34^. Overall, participants were open to learning about testicular disorders and recommended interventions that are brief, interactive, simple, and light-hearted rather than funny/cheeky ^34^. Thornton warned against the use of “cheeky” humour and puns as these can be potentially offensive and ineffective ^35^. Another factor worth considering in health promotion intervention design and delivery is men’s literacy and health literacy levels. A meta-narrative systematic review of 31 studies exploring men’s information-seeking behaviours in relation to cancer prevention found that younger men and those with high literacy and health literacy levels were more likely to engage with information delivered using technological means ^36^. By contrast, men who were older, belonged to ethnic minorities, and had low literacy and health literacy levels were more likely to engage with health information delivered by peers, physicians, and churches ^36^.

### Strengths and limitations

Rigour was ensured by systematically reporting this review using the PRISMA checklist. Moreover, a thorough search of electronic databases, trial registries, and reference lists was conducted, and records were independently screened by more than one reviewer to avoid omitting important records. However, the search was limited to records published between 2018 and 2023, which increases the risk of study selection bias, and only findings that were relevant to the review outcomes were discussed, which increases the risk of reporting bias. Due to heterogeneity, a meta-analysis was not plausible. Therefore, while promising interventions were identified and included in this updated review, definitive evidence regarding effectiveness cannot be determined.

## Conclusions

The present updated review has implications for research and clinical practice, which should be considered carefully in light of the review limitations. From a research perspective, there is a need for population-level health interventions to promote men’s awareness of testicular disorders. This could be achieved through considering the information needs and the preferred learning strategies of at-risk age groups, while accounting for sociodemographic variations within these groups ^34^. It is also essential to factor in diseases other than TC (these were underexplored in the reviewed literature), and to conduct rigorous high-quality studies capturing the longitudinal impact of the interventions on behaviours and potentially on clinical outcomes such as stage at diagnosis, treatments received, and survival rates. Examples include but are not limited to: multimedia campaigns, virtual and augmented reality interventions, gaming technologies, mobile apps, and interactive websites.

The use of theory in intervention design and delivery is key, since interventions with a theoretical underpinning are more likely to achieve the desired outcomes, particularly when there is congruence between the assumptions of the theory and those of the proposed intervention ^37^. An example is the Health Belief Model, which was used in two of the reviewed studies ^25, 26^. Another example is the Preconscious Awareness to Action Framework, a novel theoretical framework used by Saab *et al*. ^27^ to raise testicular awareness and promote early help-seeking for testicular symptoms.

From a practical standpoint, clinicians involved in health promotion are encouraged to direct men to resources where information on testicular disorders is freely and readily accessible. Given the scarcity of high-quality evidence to support scheduled TSE, lack of consensus regarding monthly TSE, clinicians should promote “testicular awareness” by encouraging men to become familiar with the look and feel of their own testes, to know which signs and symptoms to look for, and to seek prompt medical attention for symptoms of concern ^8, 30^.

## Data Availability

No data is associated with this article. Zenodo: PRISMA Checklist for "Promoting men's awareness, self-examination, and help-seeking for testicular disorders: a systematic review of interventions", https://doi.org/10.5281/zenodo.8407914 ^38^ Data are available under the terms of the Creative Commons Attribution 4.0 International license (CC-BY 4.0).

## References

[1] Manecksha RP Fitzpatrick JM : Epidemiology of testicular cancer. *BJU Int.* 2009;104(9 Pt B):1329–1333. 10.1111/j.1464-410X.2009.08854.x 19840008

[2] Rosen A Jayram G Drazer M : Global trends in testicular cancer incidence and mortality. *Eur Urol.* 2011;60(2):374–379. 10.1016/j.eururo.2011.05.004 21612857

[3] Albers P Albrecht W Algaba F : Guidelines on Testicular Cancer: 2015 Update. *Eur Urol.* 2015;68(6):1054–68. 10.1016/j.eururo.2015.07.044 26297604

[4] Hanna NH Einhorn LH : Testicular cancer--discoveries and updates. *N Engl J Med.* 2014;371(21):2005–2016. 10.1056/NEJMra1407550 25409373

[5] Wampler SM Llanes M : Common scrotal and testicular problems. *Prim Care.* 2010;37(3):613–626. 10.1016/j.pop.2010.04.009 20705202

[6] Srinath H : Acute scrotal pain. *Aust Fam Physician.* 2013;42(11):790–792. 24217099

[7] Bayne CE Villanueva J Davis TD : Factors Associated with Delayed Presentation and Misdiagnosis of Testicular Torsion: A Case-Control Study. *J Pediatr.* 2017;186:200–204. 10.1016/j.jpeds.2017.03.037 28427778

[8] Saab MM Landers M Hegarty J : The Preconscious Awareness to Action Framework: An Application to Promote Testicular Awareness. *Nurs Res.* 2018;67(2):169–176. 10.1097/NNR.0000000000000268 29489637

[9] Rovito MJ Leone JE Cavayero CT : "Off-Label" Usage of Testicular Self-Examination (TSE): Benefits Beyond Cancer Detection. *Am J Mens Health.* 2018;12(3):505–513. 10.1177/1557988315584942 25990509 PMC5987946

[10] Saab MM Landers M Hegarty J : Testicular Cancer Awareness and Screening Practices: A Systematic Review. *Oncol Nurs Forum.* 2016;43(1):E8–E23. 10.1188/16.ONF.E8-E23 26679456

[11] Roy RK Casson K : Attitudes Toward Testicular cancer and Self-Examination Among Northern Irish Males. *Am J Mens Health.* 2017;11(2):253–261. 10.1177/1557988316668131 27645516 PMC5675290

[12] Saleem D Muneer S Younus Khan RF : Knowledge, Attitude and Practices Regarding Benign Testicular Disorders in the Educated Young Men of Pakistan. *Cureus.* 2017;9(8): e1563. 10.7759/cureus.1563 29057175 PMC5640388

[13] Yap LC Keenan R Khan J : Parental awareness of testicular torsion amongst Irish parents. *World J Urol.* 2018;36(9):1485–1488. 10.1007/s00345-018-2269-8 29594530

[14] Ilic D Misso ML : Screening for testicular cancer. *Cochrane Database Syst Rev.* 2011; (2): CD007853. 10.1002/14651858.CD007853.pub2 21328302

[15] U.S. Preventive Services Task Force: Screening for testicular cancer: U.S. Preventive Services Task Force reaffirmation recommendation statement. *Ann Intern Med.* 2011;154(7):483–486. 10.7326/0003-4819-154-7-201104050-00006 21464350

[16] Rovito MJ Manjelievskaia J Leone JE : From ‘D’ to ‘I’: A critique of the current United States preventive services task force recommendation for testicular cancer screening. *Prev Med Rep.* 2016;3:361–366. 10.1016/j.pmedr.2016.04.006 27419037 PMC4929233

[17] McGuinness LA Obeidat S Hickerton B : Has increasing public health awareness influenced the size of testicular tumours among adult populations over the last 40 years? *J Public Health (Oxf).* 2017;39(1):90–94. 10.1093/pubmed/fdw014 26944075

[18] Aberger M Wilson B Holzbeierlein JM : Testicular self-examination and testicular cancer: a cost-utility analysis. *Cancer Med.* 2014;3(6):1629–1634. 10.1002/cam4.318 25103095 PMC4298389

[19] Saab MM Landers M Hegarty J : Promoting Testicular Cancer Awareness and Screening: A Systematic Review of Interventions. *Cancer Nurs.* 2016;39(6):473–487. 10.1097/NCC.0000000000000333 26859280

[20] Saab MM Landers M Hegarty J : Males’ Awareness of Benign Testicular Disorders: An Integrative Review. *Am J Mens Health.* 2018;12(3):556–566. 10.1177/1557988315626508 26783155 PMC5987954

[21] Garner P Hopewell S Chandler J : When and how to update systematic reviews: consensus and checklist. *BMJ.* 2016;354: i3507. 10.1136/bmj.i3507 27443385 PMC4955793

[22] Page MJ McKenzie JE Bossuyt PM : The PRISMA 2020 statement: an updated guideline for reporting systematic reviews. *Int J Surg.* 2021;88: 105906. 33789826 10.1016/j.ijsu.2021.105906

[23] Moyer A Schneider S Knapp-Oliver SK : Published versus unpublished dissertations in psycho-oncology intervention research. *Psychooncology.* 2010;19(3):313–317. 10.1002/pon.1561 19353515 PMC2832099

[24] Hong QN Gonzalez-Reyes A Pluye P : Improving the usefulness of a tool for appraising the quality of qualitative, quantitative and mixed methods studies, the Mixed Methods Appraisal Tool (MMAT). *J Eval Clin Pract.* 2018;24(3):459–467. 10.1111/jep.12884 29464873

[25] Akcali K Tastan S : The effects of flipped classroom model on knowledge, behaviour and health beliefs on testicular cancer and self-examination: a randomized controlled trial study. *Health Educ Res.* 2023;38(3):230–240. 10.1093/her/cyad007 36843567 PMC10655630

[26] Sagir FN Altinel B : Effects of information provided to university students through an educational brochure on health beliefs and testicular self-examination. *J Cancer Educ.* 2023;38(2):632–638. 10.1007/s13187-022-02166-8 35486360

[27] Saab MM Landers M Cooke E : Enhancing Men’s Awareness of Testicular Disorders Using a Virtual Reality Intervention: A Pre-Post Pilot Study. *Nurs Res.* 2018;67(5):349–358. 10.1097/NNR.0000000000000303 30059354

[28] Shenbagapraba N Roslin S Thivya N : Effectiveness of video assisted teaching on testicular self examination. *Medico Legal Update.* 2020;20(4):550–552. 10.37506/mlu.v20i4.1875

[29] Waheed M Luqman MS Bhatti UU : Intervene to Improve: Awareness of Testicular Self-Examination and Testicular Cancer Among Male Patients at a Tertiary Care Hospital in Lahore, Pakistan. *Cureus.* 2023;15(1): e33838. 10.7759/cureus.33838 36819420 PMC9931380

[30] Saab MM Hegarty J Landers M : Testicular awareness: the what, the why, and the how. *Int J Mens Soc Community Health.* 2019;2(1):e1–e10. 10.22374/ijmsch.v2i1.16

[31] Nasrallah P Nair G Congeni J : Testicular health awareness in pubertal males. *J Urol.* 2000;164(3 Pt 2):1115–1117. 10.1016/S0022-5347(05)67265-5 10958755

[32] Congeni J Miller SF Bennett CL : Awareness of genital health in young male athletes. *Clin J Sport Med.* 2005;15(1):22–26. 10.1097/00042752-200501000-00005 15654187

[33] Saab MM Landers M Hegarty J : Exploring awareness and help-seeking intentions for testicular symptoms among heterosexual, gay, and bisexual men in Ireland: A qualitative descriptive study. *Int J Nurs Stud.* 2017;67:41–50. 10.1016/j.ijnurstu.2016.11.016 27915088

[34] Saab MM Landers M Hegarty J : Exploring men's preferred strategies for learning about testicular disorders inclusive of testicular cancer: A qualitative descriptive study. *Eur J Oncol Nurs.* 2017;26:27–35. 10.1016/j.ejon.2016.11.001 28069149

[35] Thornton CP : Best Practice in Teaching Male Adolescents and Young Men to Perform Testicular Self-Examinations: A Review. *J Pediatr Health Care.* 2016;30(6):518–527. 10.1016/j.pedhc.2015.11.009 26778347

[36] Saab MM Reidy M Hegarty J : Men's information-seeking behavior regarding cancer risk and screening: A meta-narrative systematic review. *Psychooncology.* 2018;27(2):410–419. 10.1002/pon.4506 28728212

[37] Michie S Johnston M Francis J : From theory to intervention: Mapping theoretically derived behavioural determinants to behaviour change techniques. *Appl Psychol.* 2008;57(4):660–680. 10.1111/j.1464-0597.2008.00341.x

[38] Saab MM Davoren MP Murphy A : PRISMA Checklist for "Promoting men's awareness, self-examination, and help-seeking for testicular disorders: a systematic review of interventions" (Version 1). *Zenodo.* 2023. 10.5281/zenodo.8407914 PMC6973532 32002508

