# Supplementary material for: Promoting men’s awareness, self-examination, and help-seeking for testicular disorders: a systematic review of interventions
Source: HRB Open Res. 2023 Oct 13;1:16. Originally published 2018 May 14. [Version 3] doi: 10.12688/hrbopenres.12837.3 (PMC6973532; doi:10.12688/hrbopenres.12837.3)
Supplement: Peer review history

# Reviewer report: Reviewer response for version 2

**DOI:** 10.21956/hrbopenres.13917.r26303

**Published:** 2018-08-22

**Authors:** Catherine Hayes, Darach O'Ciaradh

**Affiliations:** 1 Department of Public Health and Primary Care, Trinity College Dublin, Dublin, Ireland; 2 Trinity College Dublin, Dublin, Ireland

The revisions undertaken by the authors have systematically addressed the concerns of the referees in a satisfactory manner.

Reviewer Expertise:

Public health

We confirm that we have read this submission and believe that we have an appropriate level of expertise to confirm that it is of an acceptable scientific standard.

# Reviewer report: Reviewer response for version 1

**DOI:** 10.21956/hrbopenres.13899.r26279

**Published:** 2018-06-28

**Authors:** Catherine Hayes, Darach O'Ciaradh

**Affiliations:** 1 Department of Public Health and Primary Care, Trinity College Dublin, Dublin, Ireland; 2 Trinity College Dublin, Dublin, Ireland

This Systematic Review has focused on assessing evidence for effectiveness of interventions to promote awareness of TSE and testicular disease and mens intentions around seeking help.

 Both referees (Public Health Physician and General Practitioner) agree that it is difficult to interpret this systematic review of effectiveness of measures to increase awareness of testicular disease self-examination when the effectiveness of testicular self-examination is itself unproven. As it does not establish the evidence for TSE it is difficult to promote TSE in a vacuum.

 It would aid the study if the rationale / justification for doing a Systematic Review in this context was more clearly explained. Also why a review is justified given the recent publication of previous reviews by two of the paper authors which are three of the five papers included in the review.

 The review itself is conducted with rigour using appropriate study selection and data extraction criteria and tools to assess quality.

 The conclusions would need to be modified as the additional evidence found in the systematic review is weak with small sample sizes and relatively poor quality studies.

Reviewer Expertise:

Public health

We confirm that we have read this submission and believe that we have an appropriate level of expertise to confirm that it is of an acceptable scientific standard, however we have significant reservations, as outlined above.

# Response

**Published:** 2018-07-03

**Authors:** Mohamad Saab

**Affiliations:** University College Cork, Ireland

The authors would like to thank the referees for taking the time to review the manuscript and for their valuable insights and feedback. Below are our responses to the referees’ queries. All changes in the second version of the manuscript were made using the “Track Changes” feature. We hope that the changes made are in line with the referees’ comments and recommendations.

*Both referees (Public Health Physician and General Practitioner) agree that it is difficult to interpret this systematic review of effectiveness of measures to increase awareness of testicular disease self-examination when the effectiveness of testicular self-examination is itself unproven. As it does not establish the evidence for TSE it is difficult to promote TSE in a vacuum.*

- **Thank you for pointing this out. This is highlighted in the discussion under summary of evidence, 3 ^rd^ paragraph in the first version of this paper. We made sure to reiterate this in the introduction in order to highlight the controversy surrounding TSE (see the 5 ^th^ paragraph of the introduction). We did also highlight the concept of testicular awareness which we developed and tested as an alternative to scheduled TSE (see the last paragraph of the conclusion)**

*It would aid the study if the rationale / justification for doing a Systematic Review in this context was more clearly explained.*

- **Despite men’s lack of awareness of BTDs and their intentions to delay help-seeking for symptoms of testicular disease, none of our two previous reviews included studies that aimed at promoting men’s awareness of BTDs and/or increasing their intentions to seek help for testicular symptoms. This was one of the key reasons for updating our two reviews (see the last paragraph of the introduction)**

*The review itself is conducted with rigour using appropriate study selection and data extraction criteria and tools to assess quali*ty.

- **Thank you**

*Why a review is justified given the recent publication of previous reviews by two of the paper authors which are three of the five papers included in the review.*

- **Due to the anticipated dearth of literature on testicular disorders, structured reviews of experimental studies were considered for inclusion prior to conducting the search (see the last paragraph under objectives).**
- **The reviews by two of the authors were identified in the database search and were deemed eligible for inclusion by two independent reviewers. **
- **As for the timeframe for updating the reviews, there is no gold standard for the frequency of updating structured reviews. However, biennial review updates are recommended by the Cochrane Library (see** **http://www.centrocochranedobrasil.com.br/cms/attachments/article/43/Handbook%205.1%20Updating.pdf** **)**

*The conclusions would need to be modified as the additional evidence found in the systematic review is weak with small sample sizes and relatively poor quality studies.*

- **The limitations and conclusions were edited accordingly in order to highlight the low level of evidence, the poor methodological quality, and the underpowered experimental studies. **

Thank you

Kind regards,

Mohamad

# Reviewer report: Reviewer response for version 1

**DOI:** 10.21956/hrbopenres.13899.r26218

**Published:** 2018-05-30

**Authors:** Eileen Furlong

**Affiliations:** 1 University College Dublin, Dublin, Ireland

The aim of this systematic review was to evaluate the effectiveness of studies promoting men’s knowledge and awareness of testicular disorders and/or self-examination, behaviours and/or intentions to examine their testes, and help-seeking behaviours and/or intentions for testicular symptoms.  

 A systematic search strategy for retrieval of appropriate evidence corresponds to the aim of the study and is appropriately outlined in narrative and in table format. 

 The methodologies of the five selected papers, along with the strengths and weaknesses of the evidence used to generate the evidence was critiqued using quality and *level of evidence assessment tool* for experimental studies and *AMSTAR 2 *measurement for structured reviews.  

 This systematic review demonstrates the process and skills in the critical appraisal and synthesis of the research evidence.

Are the rationale for, and objectives of, the Systematic Review clearly stated?

Yes

Is the statistical analysis and its interpretation appropriate?

Yes

Are sufficient details of the methods and analysis provided to allow replication by others?

Yes

Are the conclusions drawn adequately supported by the results presented in the review?

Yes

Reviewer Expertise:

PI in an FP7 RCT on eHealth; grounded theory. Symptom management, cancer care, children, advanced practice

I confirm that I have read this submission and believe that I have an appropriate level of expertise to confirm that it is of an acceptable scientific standard.

# Response

**Published:** 2018-05-30

**Authors:** Mohamad Saab

**Affiliations:** University College Cork, Ireland

Dear Eileen,

 On behalf of the co-authors, I would like to thank you for taking the time to review and comment on this systematic review.

 Kind regards,

 Mohamad
